# Genomics of lipid-laden human hepatocyte cultures enables drug target screening for the treatment of non-alcoholic fatty liver disease

**DOI:** 10.1186/s12920-018-0438-7

**Published:** 2018-12-14

**Authors:** Stephanie Breher-Esch, Nishika Sahini, Anna Trincone, Christin Wallstab, Jürgen Borlak

**Affiliations:** 1 Centre for Pharmacology and Toxicology, Hannover Medical School, Carl-Neuberg-Str. 1, 30625 Hannover, Germany; 2 Institute of Biochemistry, Charité - University Medicine Berlin, Charitéplatz 1, 10117 Berlin, Germany

**Keywords:** Non-alcoholic fatty liver disease (NAFLD), Transcriptomics, LD-associated proteins, Drug targets; nuclear receptor/PPARs, TNFα

## Abstract

**Background:**

Non-alcoholic fatty liver disease (NAFLD) is a major health burden in need for new medication. To identify potential drug targets a genomic study was performed in lipid-laden primary human hepatocyte (PHH) and human hepatoma cell cultures.

**Methods:**

PHH, HuH7 and HepG2 hepatoma cell cultures were treated with lipids and/or TNFα. Intracellular lipid load was quantified with the ORO assay. The Affymetrix HG-U133+ array system was employed to perform transcriptome analysis. The lipid droplet (LD) growth and fusion was determined by fluorescence microscopy. LD associated proteins were imaged by confocal immunofluorescence microscopy and confirmed by Western immunoblotting. Bioinformatics defined perturbed metabolic pathways.

**Results:**

Whole genome expression profiling identified 227, 1031 and 571 significant regulated genes. Likewise, the combined lipid and TNFα treatment of PHH, HuH7 and HepG2 cell cultures revealed 154, 1238 and 278 differentially expressed genes. Although genomic responses differed among in-vitro systems, commonalities were ascertained by filtering the data for LD associated gene regulations. Among others the LD-growth and fusion associated cell death inducing DFFA like effector C (CIDEC), perilipins (PLIN2, PLIN3), the synaptosome-associated-protein 23 and the vesicle associated membrane protein 3 were strongly up-regulated. Likewise, the PPAR targets pyruvate-dehydrogenase-kinase-4 and angiopoietin-like-4 were up-regulated as was hypoxia-inducible lipid droplet-associated (HILPDA), flotilin and FGF21. Their inhibition ameliorates triglyceride and cholesterol accumulation. TNFα treatment elicited strong induction of the chemokine CXCL8, the kinases MAP3K8, MAP4K4 and negative regulators of cytokine signaling, i.e. SOCS2&SOCS3. Live cell imaging of DsRED calreticulin plasmid transfected HuH7 cells permitted an assessment of LD growth and fusion and confocal immunofluorescence microscopy evidenced induced LD-associated PLIN2, CIDEC, HIF1α, HILPDA, JAK1, PDK4 and ROCK2 expression. Notwithstanding, CPT1A protein was repressed to protect mitochondria from lipid overload. Pharmacological inhibition of the GTPase-dynamin and the fatty acid transporter-2 reduced lipid uptake by 28.5 and 35%, respectively. Finally, a comparisons of in-vitro/NAFLD patient biopsy findings confirmed common gene regulations thus demonstrating clinical relevance.

**Conclusion:**

The genomics of fat-laden hepatocytes revealed LD-associated gene regulations and perturbed metabolic pathways. Immunofluorescence microscopy confirmed expression of coded proteins to provide a rationale for therapeutic intervention strategies. Collectively, the in-vitro system permits testing of drug candidates.

**Electronic supplementary material:**

The online version of this article (10.1186/s12920-018-0438-7) contains supplementary material, which is available to authorized users.

## Background

Non-alcoholic fatty liver disease (NAFLD) is a major health burden and characterized by intrahepatic lipid droplets of different size. An essential criterion for the histological definition of NAFLD is the presence of lipid droplets in > 5% of hepatocytes [1]. The prevalence of fatty liver disease ranges between 40 and 90% in Western countries and is about 75% among obese individuals [2]. NAFLD is hallmarked by an imbalance in lipid homeostasis, abnormalities in mitochondrial metabolism, endoplasmic reticulum (ER) stress, impaired glucose tolerance, dyslipidemia and cardiovascular disease. Note, clinical evidence suggests NAFLD and the metabolic syndrome to be potentially linked with insulin resistance being a prerequisite for its development [3–5]. Therefore, a complex relationship exists between obesity, insulin resistance and NAFLD while the diagnostic criteria for the metabolic syndrome encompass a range of abnormalities, i.e. BMI > 30, impaired fasting glucose, impaired glucose tolerance, Type 2 diabetes, high blood pressure, elevated plasma triglycerides and HDL cholesterol as well as increased urinary albumin excretion [6]. Collectively, NAFLD and the metabolic syndrome involve a spectrum of conditions and when accompanied by inflammation results in liver fibrosis and cirrhosis that is a possible route to hepatocellular carcinoma (HCC) [7, 8]. Drug treatment may also induce hepatic steatosis or even exacerbates pre-existing NAFLD.

Given that over-nutrition can develop into obesity and obesity is a common cause of NAFLD, it is justified to briefly summarize lipid uptake. Under the influence of intestinal enzymes, e.g. lipase and amylase, dietary lipids and carbohydrates are digested to monoglycerides, fatty acids, glucose, fructose and monosaccharides. Prior to systemic circulation, the nutrients pass the intestinal barrier and in the case of lipid/cholesterol uptake chylomicrons are formed in enterocytes and are released via the lymphatic capillaries into the systemic circulation such as the portal and/or jugular vein. Chylomicrons are then modified by lipoprotein lipase to produce chylomicron remnants which are taken up by the liver and are further processed in hepatocytes for the synthesis of triglycerides (TG). Free fatty acids in the circulation are mostly bound to albumin. Notwithstanding, hepatocytes are also major sites for de novo lipid synthesis from glucose where pyruvic acid is formed through glycolysis and further processed to acetyl CoA, i.e. a key and versatile intermediate that enters a series of reactions to produce triacylglycerols [9]. Alternatively, acetyl CoA derived from glucose metabolism or mitochondrial fatty acid-β-oxidation is processed in the citric acid cycle for energy production, i.e. ATP synthesis. Mitochondrial glucose and fatty acid metabolism can be a major source for reactive oxygen species (ROS) that is detoxified via superoxide dismutase, catalase and glutathione dependent reactions. However, in hepatic steatosis enhanced lipid metabolism is associated with increased ROS production that might exceed the cellular capacity of ROS detoxification and as a result causes lipid peroxidation and mitochondrial dysfunction to lower considerably the energy output to peripheral tissues [10]. In the long term and through a vicious cycle that involves the peptide hormone adiponectin, fat-laden hepatocytes engage in lipogenesis rather than lipid metabolism, and lipid removal via VLDL secretion becomes insufficient to reduce effectively the hepatic intracellular lipid burden.

Recently, we summarized the molecular pathophysiology of lipid droplet formation in hepatocytes [4], and targeting the unfolded protein response offers new possibilities in the treatment of NAFLD. Specifically, hepatic steatosis induces ER stress and as a result the unfolded protein response [11]. This pathway involves complex signaling cascades aimed at restoring cellular homeostasis. However, prolonged hepatic steatosis disrupts the ER and secretory pathway homeostasis and is predominantly associated with insulin resistance, inflammation and apoptosis [12, 13]. Moreover, ectopic LD formation perturbs physical and functional links between mitochondria and ER to influence hepatocellular metabolism [14]. Several models have been proposed [15, 16] that support the biogenesis of lipid droplets from the ER. It will be worthwhile to investigate the role of oxidative and ER stress for a better understanding of the pathogenesis of fatty liver disease.

There is unmet need for new medications to treat fatty liver disease. Therefore, our study aimed at identifying perturbed metabolic pathways and LD-associated proteins as potential drug targets in fat-laden human hepatocyte cultures. We explored two different routes in the budding and growth of lipids droplets, i.e. fatty acid treatment of cell cultures and ER stress induced by dithiothreitol. We focused on lipid droplet associated gene regulations as putative drug target and equally considered adaptive responses to hepatic steatosis. Note, depending on the cell type the lipid droplet can be dressed with hundreds of proteins [17] and several of these proteins are interesting drug targets for the treatment of NAFLD. This includes Plin2, i.e. a key regulator of the unfolded protein response and ER stress [18]. Besides, the fusion of smaller lipid droplets (LD) to larger ones causes macrovesicular steatosis and eventually ballooned hepatocytes to worsen the condition of NAFLD. Thus, LD-fusion and its association with the endoplasmic reticulum were examined by live cell imaging and immunofluorescence microscopy in DsRED calreticulin plasmid transfected human hepatoma cells. As primary human hepatocytes are not routinely available and may involve donor specific responses, the genomics of lipid-laden human hepatoma cells HuH7 and HepG2 were also compared to findings obtained with primary human hepatocyte cultures. Moreover, non-alcoholic steatohepatitis (NASH) is characterized by inflammation. Typically serum levels of the cytokine TNFα are increased in NASH patients and are shown to correlate with histologic scores of liver injury [19]. Therefore, we investigated the genomic responses of steatotic hepatocyte cultures to TNFα treatment.

Collectively, transcriptomics of fat-laden hepatocytes informed on NAFLD response genes and enabled the search for mechanistically linked and lipid droplet associated gene regulations. The study revealed putative and established drug targets with some targets being validated by confocal immunofluorescence microscopy and Western immunoblotting. Furthermore, clinical relevance of in-vitro NAFLD response genes was established by confirming their regulation in patient liver biopsies. Altogether, the employed research strategy permits the search for novel LD-associated targets for the pharmacological treatment of NAFLD and to test drug candidates to broaden the perspective of new medications.

## Methods

Liver specimens from *N* = 6 patients undergoing elective surgery for colorectal liver metastasis were used to isolate hepatocytes for their culture as detailed below. The basic patient characteristics are given in Additional file 1: Table S1. All tissue donors gave informed consent for experimental use of clinical data and liver specimen prior to surgery. Ethical approval for the protocol and use of liver samples was obtained from the Ethics Committee of the Hannover Medical School (Tr/L, 2499 and Tr/L, 466 31309). Additionally, the human hepatoma cell lines HepG2 and HuH7 are established cell lines and are available through the American type culture collection (ATCC) and the Japanese Collection of Research Bioresources Cell Bank (JCRB0403) of the National Institutes of Biomedical Innovation, Health and Nutrition, Japan.

### Primary human hepatocyte cell culture

Healthy specimens of liver section material were immediately transferred in ice cold physiologic saline solution to the laboratory, and hepatocytes were isolated as described previously [20, 21]. Vessels visible on the cut surface were cannulated and the perfusion was initiated with 200 mL of an ethylene glycol tetraacetic acid (EGTA) -containing HEPES buffer at pH 7.4 and 37 °C. Subsequently, the perfusion was continued with 200 mL HEPES buffer. Thereafter, extracellular matrix degradation was achieved by perfusion with 200 mL of a HEPES buffer containing collagenase (Liberase CI, Roche, Germany) and calcium chloride dihydrate at 37 °C. The enzyme containing perforate solution was recirculated. Following perfusion, the liver capsule was carefully removed, and cells were liberated by gentle shaking of the liver specimen in ice cold buffer containing Hanks buffered salt HEPES and bovine serum albumin. The resulting cell suspension was filtered through a nylon mesh and washed three times in buffer at 4 °C. Viability of the hepatocytes was assessed by trypan blue exclusion and ranged between 78 and 94%.

Primary human hepatocytes (PHH) were cultured in 6-well plates (Techno Plastic Products AG, Switzerland) in a collagen sandwich as described previously [21, 22] for up to 7 days. Approximately 1, 5 million hepatocytes were seeded per well and after attachment the non-adherent cells were removed and a second layer of collagen was applied on top of the cells. The PHHs were cultured with supplemented William’s E culture medium (Lonza GmbH, Germany) at 37 °C with 100% humidity and 5% CO2. The cell culture medium was changed daily and morphologically inspected by phase contrast microscopy.

### HepG2 and HuH7 cell culture

The human hepatoma cell lines HuH7 and HepG2 were cultured in DMEM supplemented with 10% fetal calf serum, 1% glutamine and 1% pen- strep at 37 °C with 100% humidity and 5% CO2. The cell culture media and reagents were purchased from Gibco, USA.

### Cell treatment

At around 80% confluence cells were treated with 0.5 mM of an equimolar mixture of palmitic and oleic acid (PA/OA) and/or TNFα (5 ng/ml) for either 72 h or 7 days. The final DMSO concentration in control and PA/OA treated cell cultures was 0.5% *v*/v, and culture medium was changed daily.

### ER stress induced LD formation

HuH7 cells were seeded at 1 × 10^5^ in a 24 well plate and transfected with ds RED calreticulin for 24 h using PolyFect transfection reagent from Qiagen according to the manufacturer’s recommendations. Note, the ds RED calreticulin plasmid is the kind gift of Professor Naim, Institute of Biochemistry, University of Veterinary Medicine Hannover. Post transfection, cells were treated for 24 h with 10 μM DTT (dithiotriol) and this reagent protects sulfhydryl groups of proteins against oxidation. The lipids were visualized with the fluorescent dye monodansyl pentane (MDP) for 15 min, and live cell images were captured using the TiE Nikon microscope. The images were analyzed with the NIS elements software version 4.13.

As mentioned above, the HuH7 cells were transfected with the DsRED calreticulin plasmid and treated with 1:1 equal mixture of 0.5 mM oleic (OA) and palmitic (PA) acid for 24 h. Post treatment, cells were fixed with 4% formalin and stained with MDP for 15 min and the coverslips were mounted using Mowiol. The images were captured using the Nikon Ti-E microscope.

### Imaging of intracellular lipid load

Hepatocyte cultures were treated with 1:1 mixture of 0.5 mM PA/OA for 1 h, 2 h, 4 h, 6 h, 24 h, 48 h and 72 h. The cells were fixed with a 4% formalin solution, 60% isopropanol and stained with oil red O (ORO) according to the manufacturer's recommendation (Sigma Aldrich, Germany). The images were captured at a magnification of 20x by microscopy using appropriate filters for the ORO and DAPI nuclei stain.

### Intracellular lipid quantification

Calibrates consisting of 0, 10, 25, 50 and 100 μg/ml (PA/PO) were prepared and placed into disposable cuvettes. ORO was added and the absorbance was measured at 520 nm in a spectrophotometer according to the manufacturer’s recommendation. A standard curve was fitted by plotting calibrates against absorbance readings. Typically, a correlation coefficient of R^2^ > 0.975 was obtained. To determine intracellular lipid content the media of lipid-laden hepatocyte was removed and the ORO staining of cell cultures was performed as described above. Excess dye was removed by repeated washing. Subsequently, intracellular ORO stained lipids are extracted by adding 4% Igepal CA-630 in 1 ml of isopropanol. The solution was measured in a spectrophotometer at 520 nm to quantify the concentration of ORO against the standard curve.

### Inhibition of FA transporters dynamin and FATP2

HepG2 cell cultures (1.2 × 105 cells/well) were incubated at 37 °C with DMEM (K-), DMSO (K+), PA/OA, PA/OA + 80 μM dynamin blocker (Dynasore, Abcam, UK) and/or PA/OA and 9 μM FATP2 blocker (CB16.2). The steatotic cell cultures were treated with dynasore and/or CB16.2 for 1 h; the FATP2 blocker was kindly provided by Prof. Concetta C. DiRusso, University of Nebraska [23]. For the dynamin assay, culture medium without serum was used as serum inhibits the effect of the Dynasore while studies with CB16.2 to block FATP2 were conducted in the presence of serum.

Post treatment, cells were harvested, washed three times with PBS and fixed with 4% formaldehyde. After incubation at room temperature for 20 min, the cells were washed with deionized water and treated with 60% isopropanol for 3–5 min and finally suspended in oil red O (ORO) stain (Sigma Aldrich, Germany) and incubated at room temperature for 10 min. After staining, the cells were washed several times with deionized water to remove excess stain and re-suspended in 100 μl running tap water. The ORO stain was imaged with Texas Red filter (excitation562/emission624) by phase contrast fluorescence and light microscopy using the Nikon TiE microscope. Furthermore, quantification of the intracellular lipid was done as described before,  and statistical testing involved a t-test and was considered significant at *p* < 0.05.

### Immunofluorescence phase contrast live cell imaging

The human hepatoma cells HuH7 were incubated for 24 h with 1:1 mixtures of 0.5 mM oleic and palmitic acid. Additionally, HepG2 cells were treated with a 0.5 mM mixture of PA/OA for 3 h and LD fusion was assayed with propranolol at 200 μM. The images were captured by phase contrast fluorescence microscopy at 40x using time-lapse z-stack (Nikon TiE) and further analyzed using the NIS elements software version 4.13. Lipid droplets were visualized with the fluorescent MDP dye.

### Immunofluorescence microscopy of PLIN2, CIDEC, CPT1A, HILPDA, HIF1α, JAK1, PDK4 and ROCK2

Perilipin 2 (PLIN2) was purchased from Antibodies Online (Aachen, Germany) (cat# ABIN112185), the cell death-inducing DFFA-like effector c (CIDEC), CPT1A, HIF1α, JAK1 and Rock 2 were obtained from Santa Cruz Biotechnology, Inc. (cat# sc-99,342, sc-139,480, sc-10,790, sc-277 and sc-5561). HILPDA was purchased from ProSci, Poway, USA (cat. 6491). Briefly, PHHs and HuH7 cell cultures were treated with 0.5 mM of an equal mixture of PA/OA for 24 h, fixed with 4% formalin and permeabilised with 0.2% TritonX100 for 5 min. The hepatocytes were incubated at room temperature in a dark chamber with primary antibody (1:200) for one hour and with the secondary antibody (1:50) for 45 min. The lipid droplets were stained with the fluorescent dye monodansylpentane at 0.1 mM for 15 min followed by mounting of the slides with Mowiol 4–88. Immunofluorescence microscopy was performed with the Nikon TiE phase contrast microscope as detailed above.

### Western blotting (WB) of PLIN2

WB experiments were done with the Trans-Blot Turbo system according to the manufacturer’s recommendations (Bio-Rad, Munich, Germany). After SDS-PAGE the gel was sandwiched within the blotting paper and the PVDF membrane and placed inside the cassette of the Trans-Blot Turbo Transfer System (Bio-Rad, Germany). Both the membrane and the blotting paper sheets were provided as ready-to-use in the Midi Format kit (Bio-Rad, Germany). Transfer of proteins was done with a protocol preset by the manufacturer, i.e. up to 25 V at constant amperage of 2.5A for 7 min. After blotting the gels were discarded and the successful transfer of proteins was confirmed by staining the membrane with Ponceau S solution (0.1% in 5% acetic acid, Sigma Aldrich, Germany). The blotting membrane was placed in a 5% milk powder solution dissolved in 1X Tris Buffered Saline + 0.1% Tween 20 (TBS-T) (TBS contains 50 mM Tris-HCl ph 7.6 and 150 mM NaCl). Membranes were gently shaken for 1 h, and this procedure was followed by 3 washings with freshly made TBS-T lasting 10 min each in agitation. Primary antibodies were diluted in a proper amount of TBS-T and incubation of the antibody with the blotting membrane was done overnight at 4 °C in agitation. Thereafter, membranes were washed 3 times with 2.5% milk in TBS-T (10 min each) and the incubation of the secondary antibody was carried out for 1 h at room temperature after diluting the antibody in 5% milk dissolved in TBS-T. Finally, 3 additional washings with TBS-T were done to remove unbound antibody.

Proteins were detected with the Clarity Western ECL Substrate kit (Bio-Rad, Germany). The kit contains two reagents that were mixed in a 1:1 ratio. The horseradish peroxidase conjugated to the secondary antibody catalyzes the reaction between luminol and hydrogen peroxide (contained in the solutions of the kits), providing chemiluminescence that can be detected by a CCD camera. Membranes were removed from the box and dried from the excess of TBS-T before adding homogeneously 800 μl of the reagents mixture. The reagent was applied for 3 min in the dark before placing the membrane inside the Chemidoc MP system (Bio-Rad, Germany) to permit detection of the band and image acquisition. Exposure time was usually set automatically by the device.

### Statistical analysis of cell culture experiments

Statistical significance testing was done with the GraphPad Prism Software (version 6.05) for Windows, GraphPad Software, La Jolla California USA, https://www.graphpad.com/). Except for the 1 h time period where a Wilcoxon matched-pairs signed rank test was used to study lipid uptake all other cell culture experiments involved a Mann Whitney test. * denotes a significant *p*-value < 0.05, ***p < 0.001 and ****p < 0.0001.

### Microarray data analysis

Total RNA was isolated with the Qiagen RNA purification kit according to the manufacturer’s instructions (Qiagen, Hilden, Germany). cRNA was prepared following the Affymetrix Gene Chip® Expression Analysis Technical Manual (Santa Clara, USA) [24] and whole genome transcript expression analysis was carried out with the Affymetrix Gene chip HG-U133 version 2.0 array [25]. Whole genome microarray data were normalized by applying the robust multi-array average (RMA) method using the Gene Expression Console software for background-adjusted and log-transformed perfectly matched individual probes. Genes annotated with unknown functions (i.e. “EST/hypothetical proteins” or “ORF of unknown functions”) were removed from the dataset. Subsequently, the data were transferred to the Transcriptome Analysis Console (TAC) and t-test was performed for statistical analysis of differentially expressed genes by comparing data from vehicle controls versus lipid treated cell cultures. Additionally, the TAC software performs the Benjamini-Hochberg procedure to determine the false discovery rate (FDR) that was controlled at an alpha level of 0.05. The FDR was considered when determining statistically significantly regulated genes at *p* < 0.05. The data were filtered for 300 genes which are considered to be mechanistically relevant in the process of lipid droplet biogenesis in hepatocytes as recently published by us [4]. The microarray data is available through the GEO Omnibus public repository (accession number: GSE122660).

## Results

### Lipid droplet formation induced by ER stress

**Fig. 1:**
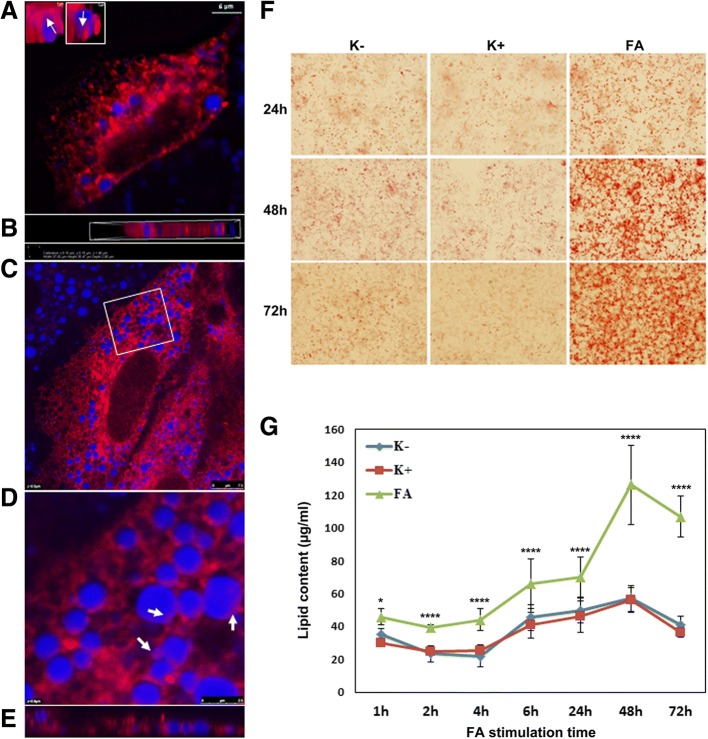
ER stress induced LD formation and quantification of intracellular hepatic lipid content in HepG2 cells. **a** & **b** Depicted are HuH7 cells transfected with ds RED calreticulin. The cultures were treated with 10 μM DTT for 24 h. The lipid droplets were stained with the fluorophore MDP (blue) and live cell images were captured with the TiE Nikon microscope. The images were analyzed with the NIS elements software version 4.13 and images in the inset are single LDs. Regions in pink (as indicated by arrows) appear to be associate with ER. The scale bar is 6 μm. Panel B is a view of panel A in x-y-z plane. **c-e** Fatty acid induced ER stress. Depicted are live cell images of HuH7 cells transfected with ds RED calreticulin. The cultures were treated with 1:1 equal mixture of oleic acid (OA) and palmitic acid (PA) for 24 h. The images were captured using a Leica SP5 confocal microscope. The scale bar is 7.5 μm. Panel D is a zoomed image of the rectangular marked region shown in panel C and panel E depicts the Y-plane of the white dotted line shown in panel D. The arrows indicate the region where LDs appear to be associated with ER. **f** Phase contrast images of intracellular lipid droplets. Shown is the time dependent growth of LDs after FA stimulation. HepG2 cells were either treated with the (K+) or without (K-) DMSO vehicle control or PA/OA (FA) for 24, 48 and 72 h. LDs are visualized with the ORO stain. Images were captured with a Nikon TiE phase contrast fluorescent microscope using the NIS elements software version 4.13. **g** Time dependent increase in intracellular lipid content of HepG2 cells treated with 1:1 mixture of 0.5 mM PA/OA for up to 72 h. Further information is given in Additional file 2: Figure S1, Additional file 3: Figure S2, Additional file 4: Figure S3. * denotes a significant *p*-value < 0.05 and ****p < 0.0001

The DsRED calreticulin plasmid was used to visualize ER. After successful transfection of HuH7 cells the cultures were treated with 10 μM DTT for 24 h. Note, dithiothreitol blocks disulfide-bond formation and causes ER stress to result in ectopic LD formation (Fig. 1a&b). The inserts depicted in Fig. 1 are images of single LDs stained with the fluorophore monodansylpentane (in blue); the region in pink indicated by arrows illustrates an apparent association with the ER as hallmarked by the DsRED fluorophore. Furthermore, the calreticulin plasmid was studied in HepG2 cell but the transfection efficiency was low (data not shown). Given that HuH7 cells are larger as compared to HepG2 cells the lipid droplet/ER association could be better visualized in the HuH7 cell line. We also investigated ectopic LD formation in cultures of primary human hepatocytes but the DTT treatment proved to be toxic and did not permit image analysis.

### Lipid droplet formation induced by fatty acid treatment

Similar results were obtained after lipid treatment of HuH7 cells. Here, LDs appeared to be associated with ER (regions in pink indicated by arrows in Fig. 1c-e). The kinetics of intracellular lipid uptake in hepatoma and primary human hepatocyte cultures was investigated; a significant difference between the DMSO vehicle and palmitate-oleate-treated cell cultures was observed. Using the oil red O (ORO) stain the lipids were quantified at 1 h, 2 h, 4 h, 6 h, 24 h, 48 h and 72 h following fatty acid treatment, and the total intracellular lipid concentration increased from 45 (1 h) to 70 μg/ml after 24 h. Figure 1f depicts phase contrast images of total intracellular hepatic lipid content visualized with the ORO stain; the kinetics of lipid uptake is depicted in Fig. 1g. When compared to the DMSO vehicle control the intracellular lipid content increased further by about 80 and 70 μg/ml at 48 and 72 h, respectively. Similar results were obtained for the HuH7 and primary human hepatocyte cultures (data not shown). See Additional file 2: Figure S1 for additional information.

### Live cell imaging of LD growth

As described previously [26, 27] LD fusion events involve transfer of lipids and other LD components from one LD to the other and may grow by expansion or coalescence. To determine LD growth HuH7 cells were treated with a 1:1 mixture of 0.5 mM oleic acid and palmitic acid for 24 h and studied by live cell imaging. Heterotypic LD fusion was observed where smaller LDs (orange arrows) fuse with larger ones (red arrows) (Additional file 3: Figure S2A-E).

Alike, steatotic HepG2 cells were treated for 3 h with propranolol to trigger LD fusion. Note previous studies have shown that the β-adrenoceptor antagonist propranolol functions as a fusogen possibly through interaction with the phospholipid monolayer [28]. As depicted in Additional file 4: Figure S3 the live cell images show a clear demarcation between the untreated and propranolol treated HepG2 cells. The fatty acid treated cells were filled with LDs and when treated with propranolol the cells show larger but fewer LDs. As a result multiple lipid droplets randomly grew in size, and the LD fusion events were captured showing mostly heterotypic fusion events with differing radii of LDs as was observed in the fatty acid treated HuH7 cells (Additional file 4: Figure S3B). The entire event was recorded for 15 min. Post 3 h of propranolol treatment the cells were apoptotic and hence could not be used to evaluate gene expression changes.

Importantly, recent research evidenced propranolol to worsen liver injury in a mouse model of NASH via activation of death pathways. Therefore, the drug should be given to NAFLD patients with caution [29].

### Inhibition of hepatic lipid uptake

Recently the DiRussco lab reported inhibition of fatty acid transport by CB16.2 (=Lipofermata) and CB5 (=Grassofermata) with an IC50 of 6.7 ± 0.6 and 6.3 ± 09 μM, respectively [30–32].

**Fig. 2:**
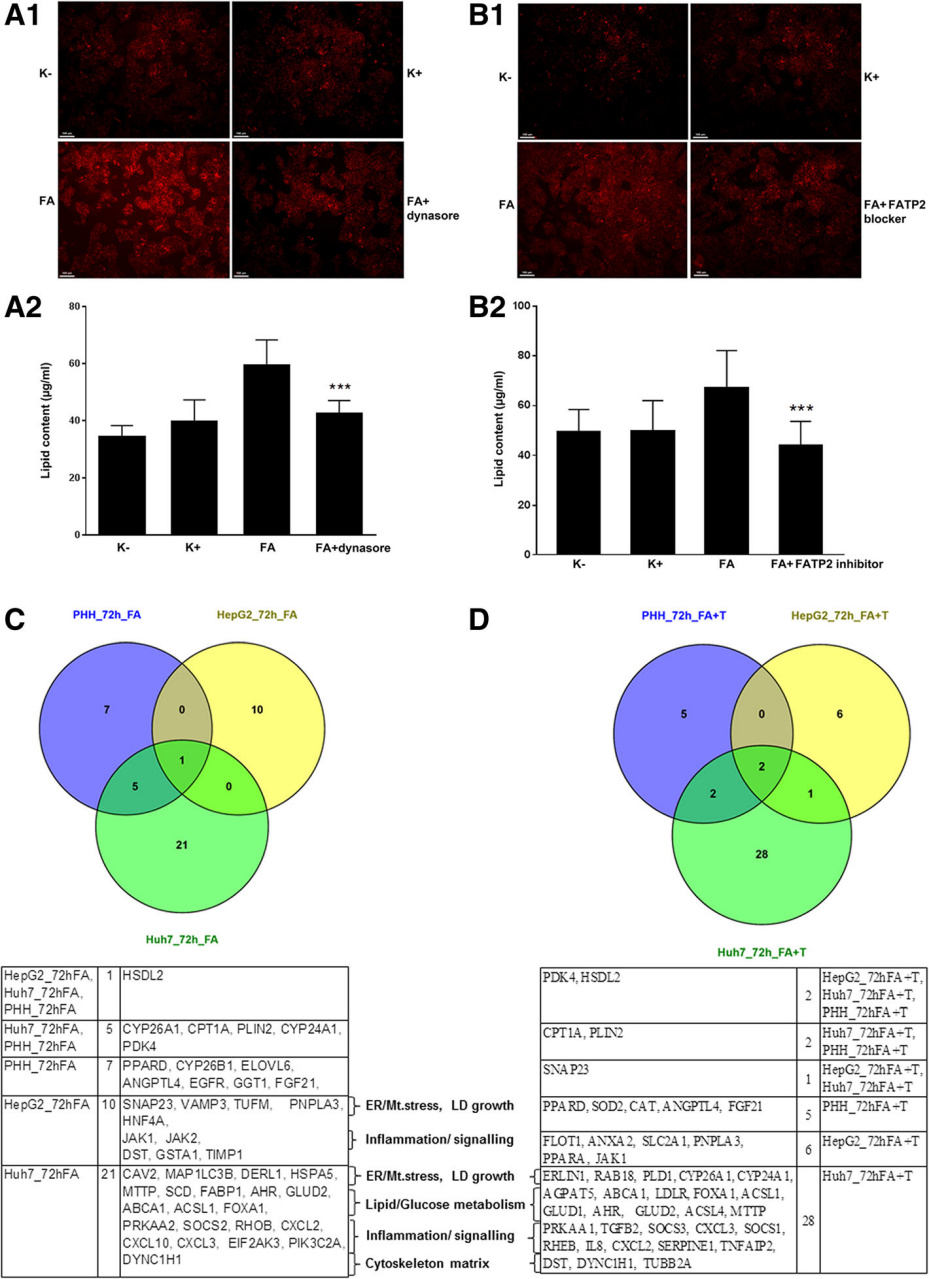
Effects of dynamin and FATP2 inhibitors on hepatic lipid uptake and Venn diagram of commonly regulated genes in steatotic primary human hepatocyte and human hepatoma cell cultures. **A1** HepG2 cell cultures were treated with either DMSO or FA or FA and dynamin (=LDLR blocker) for 1 h. The fluorescence images were captured at 10x with a Nikon TiE phase contrast microscope and analyzed with the NIS elements software version 4.13. **A2** When compared to the FA treatment alone the combined treatment of HepG2 cell cultures with FA and the dynamin inhibitor dynasore reduced significantly hepatic fatty acid uptake (*p* < 0.001). **B1** HepG2 cell cultures were treated with either DMSO or FA or FA and the FATP2 blocker CB16.1 for 1 h. The fluorescence images were captured at 10x with a Nikon TiE phase contrast microscope and analyzed with the NIS elements software version 4.13. **(B2)** When compared to the DMSO vehicle (K+) control the combined fatty acid (FA) and FATP2 inhibitor treatment reduced significantly hepatic fatty acid uptake (*p* < 0.001). The symbol K- refers to cell cultures without treatment of the DMSO vehicle control. The ORO lipid stain was observed with the Texas Red filter; the size bar refers to 100 μm. *** denotes a significant *p*-value < 0.001. **c** Depicted is a comparison of PHH, HepG2 and HuH7 cells treated with a 1:1 mixture of 0.5 mM PA/OA for 72 h. HSDL2 was commonly regulated. **d** Depicted is a comparison of PHH, HepG2 and HuH7 cells treated with a 1:1 mixture of 0.5 mM PA/OA and TNFα for 72 h. HSDL2 and PDK4 were commonly regulated

As a proof-of-concept pharmacological inhibition of the fatty acid transport protein 2 (FATP2) by CB16.2 and of dynamin, which functions as a large GTPase in vesicle transport and the regulation of endocytosis, was investigated in human hepatoma cell cultures. For this purpose HepG2 cells were treated with either media control (K-), the DMSO vehicle control (K+), with PA/OA (lipid) or with lipid and inhibitors of fatty acid uptake for 1 h. Subsequently, the intracellular lipids were stained with ORO (Fig. 2 A1 & B1), and the total lipid content was quantified in a spectrophotometer at 520 nm (Fig. 2 A2 & B2) as described in the Material and Method section. The images (Fig. 2 A1 & B1) were captured at 10x magnification by phase contrast microscopy and analyzed using the NIS elements software version 4.13.

When compared to PA/OA treated cells the intracellular lipid content was significantly reduced by 28.5 and 35% using dynasore or CB16.2, respectively. Note, both are small molecule inhibitors of dynamin and FATP2 (Fig. 2 A2 & B2). Previous studies demonstrated that treatment of Hela cells and macrophages with 80 μM dynasore blocked dynamin activity effectively and reduced total cholesterol uptake by about 15% [33]. In the present study lipid uptake in HepG2 cell cultures was also significantly inhibited by dynasore (Fig. 2 A2). It is well known that dynasore interferes with the regulation of endocytosis by blocking dynamin, i.e. a crucial molecule for clathrin-dependent coated vesicle formation [34, 35]. While long chain fatty acids (LCFA) are taken up via caveolae mediated endocytosis [36] blocking the process of endocytosis could effectively reduce influx of fatty acids into hepatocytes. Similarly, inhibition of FATP2 activity by CB16.2 resulted in 35% reduction in FA uptake of HepG2 cells (Fig. 2 B2).

### Whole genome transcriptome profiling

We investigated genomic responses to steatosis and inflammation in cultures of primary human hepatocyte and hepatoma cell lines. Altogether 227, 1031 and 571 genes were significantly regulated in PHH, HuH7 and HepG2 cell cultures, respectively after treatment with fatty acids for 72 h. Equally, the combined treatment with lipid and TNFα caused significant regulation of 154, 1238 and 278 DEGs in PHH, HuH7 and HepG2 cell cultures. Bioinformatics revealed perturbed metabolic pathways but also adaptive responses to hepatic steatosis. Subsequently, the data were filtered for DEGs considered to be mechanistically linked to lipid droplet biogenesis (Additional file 5: Table S2) [4]. Shown in Fig. 2c&d are Venn diagrams to inform on LD associated gene regulations. Specifically, hydroxysteroid dehydrogenase like 2 (HSDL2) is commonly up-regulated among the different cell culture systems after 72 h of lipid treatment. The protein functions in the transport and metabolism of fatty acids and cholesterol esters. Its dual localization in mitochondria and peroxisomes was confirmed by confocal microscopy [37]. Several genes were commonly up-regulated when at least two cell culture systems were compared (Fig. 2c) and included the carnitine palmitoyltransferase 1A (CPT1A), a rate-limiting enzyme in the ß-oxidation of long chain fatty acids. However, expression of the protein was reduced to protect mitochondria from lipid overload as evidenced by immunofluorescence microscopy (see below). Likewise, the delta-9 stearoyl-coA desaturase (SCD) was up-regulated. Inhibition of the ER localized enzyme increases insulin sensitivity and resistance to diet-induced obesity [38]. Further examples include the peroxisome proliferator-activated receptor PPARβ/δ and its targets ANGPTL4 as well as pyruvate dehydrogenase kinase, isozyme 4 (PDK4) whose expression were significantly up-regulated in cultures of primary human hepatocytes after lipid treatment for 72 h. The induction of perilipin 2 and PDK4 exemplifies the suitability of the in-vitro system to investigate drug candidates for the treatment of hepatic steatosis. Specifically, expression of perilipin 2 and PDK4 is augmented in LD biogenesis with PDK4 inhibiting the pyruvate dehydrogenase complex. Hif1α promotes PDK4 gene expression and although not significantly changed at the transcript level Hif1α protein expression was strongly induced particularly after the combined lipid and TNFα treatment (see below immunofluorescence microscopy). Therefore, mechanistic aspects in the mitochondrial pathogenesis of hepatic steatosis can be investigated. Likewise induction of the monooxygenases CYP24A1, CYP26A1 and CYP26B1 informs on retinoic and fatty acid oxidations. The regulation of the ras homolog family member B (RHOB) alerts to changes in cytokinesis and microtubule-dependent signaling and induced expression of the microsomal triglyceride transfer protein (MTTP) signifies increased cholesterol biosynthesis and triglyceride accumulation. Conversely, induction of FOXA1 highlights adaptive responses to hepatic steatosis. This transcription factor was shown to reduce lipid accumulation in human hepatocytes and to repress lipid uptake by inhibiting the fatty acid transporter FATP2 [39]. A further example of an adaptive response is the significant up-regulation of fibroblast growth factor 21. There is clear evidence for FGF21 signaling to ameliorate lipotoxicity, inflammation and to improve mitochondrial function [40].

### Combined lipid and TNFα treatment

To mimic inflammation cell cultures were treated with lipid and TNFα. Importantly, increased serum TNFα levels correlate with histologic scores of liver injury in NASH patients [19]. A comparison of the genomic responses among different cell culture systems revealed HSDL2 and PDK4 to be commonly regulated (Fig. 2d). Additionally, genes commonly up-regulated in at least two culture systems included CPT1α as well as the lipid droplet associated PLIN2 and SNAP23, the latter facilitating lipid droplet fusion. The transcriptional up-regulation of CPT1α and PPARα (HepG2, 72 h) indicate coordinate responses to influence fatty acid uptake and ß-oxidation in mitochondria. Conversely, the up-regulation of MTTP and ANGPTL4 imply increased cholesterol ester biosynthesis and triglyceride accumulation in hepatocytes. The low density lipoprotein receptor (LDLR) was also up-regulated in Huh7 treated cell cultures and induced CYP24A1 and CYP26A1 monooxygenases are part of vitamin D and retinoic acid signaling. Note induced CYP24A1 expression may provide a rational for the common observation of vitamin D deficiency in NAFLD patients [41]. Additionally, annexin A2 was up-regulated (HepG2, 72 h) and the coded protein functions in cell motility, cytoskeleton and endocytosis.

**Fig. 3:**
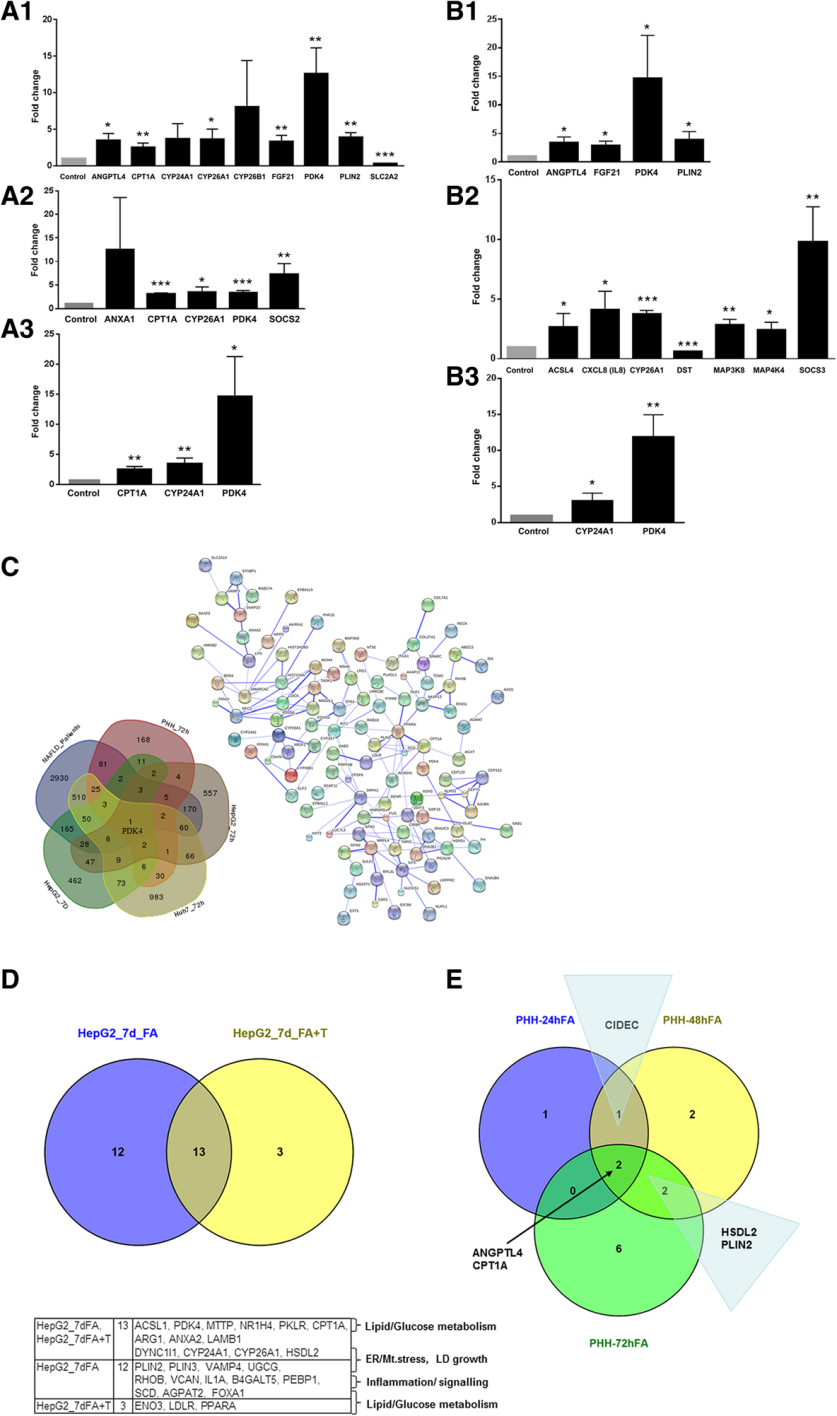
DEGs with high fold changes among lipid-laden in-vitro cultures after treatment for 72 h (PHH, HuH7) or 7d (HepG2) and Venn diagrams of commonly regulated genes in steatotic hepatocyte cultures at various time points. **a** Significantly regulated DEGs with a fold change of 3 or above in cultures of primary human hepatocyte **A1** and HuH7 cultures **A2** after lipid treatment for 72 h. **A3** Results obtained with HepG2 cultures after lipid treatment for 7d. **b** Significantly regulated DEGs with a fold change of 3 or above in cultures of primary human hepatocyte **B1** and HuH7 cultures **B2** after lipid and TNFα treatment for 72 h. **B3** Results obtained with HepG2 cultures after lipid and TNFα treatment for 7d. * denotes *p* < 0.05, ** *p* < 0.01 and *** *p* < 0.001. **c** Comparison of NAFLD patient liver biopsy findings [54] with in-vitro cell culture data. Shown is a Venn diagram; only PDK4 was commonly regulated when the different data sets were compared. Additionally a STRING network of protein-protein interactions of 108 commonly regulated genes amongst three different data sets is given. **d** Comparison of DEGs in HepG2 cells treated with a 0.5 mM mixture of oleic acid and palmitic acid and/or TNFα for 7d. Thirteen genes were commonly regulated (ACSL1, PDK4, MTTP, NR1H4, PKLR, CPT1A, ARG1, ANXA2, LAMB1, DYNC1L1, CYP24A1, CYP26A1, HSDL2). **e** The Venn diagram displays commonly regulated genes in primary human hepatocyte cultures treated with a 0.5 mM mixture of oleic acid and palmitic acid for 24 h, 48 h and 72 h. Two genes (ANGPTL4 and CPT1A) were commonly regulated at any time point. At the 24 h and 48 h time points, CIDEC was commonly regulated while PLIN2 and HSDL2 were commonly regulated genes after 48 h and 72 h

To further evaluate genomic responses genes ≥3-fold regulated genes were considered (Fig. 3, panel A and B). With primary human hepatocyte cultures the genes coding for PDK4, PLIN2, ANGPTL4 and FGF21 were commonly up-regulated after lipid and the combined lipid and TNFα treatment (Fig. 3 A1 and B1). The significant induction of PDK4 alters mitochondrial glucose and fatty acid metabolism; its activity is influenced by PPARβ/δ and HIF1α [42]. ANGPTL4 is a direct PPARβ/δ target and was shown to participate in glucocorticoid-regulated triglyceride metabolism by inhibiting lipoprotein lipase-mediated plasma triglyceride clearance [43, 44]. Previous research also demonstrated ANGPTL4 to increase cholesterol synthesis in the liver [45].

The up-regulation of fibroblast growth factor 21 is of great importance. FGF21 treatment of mice was shown to correct obesity [46] and to improve insulin sensitivity and glucose control in rodents and humans [47]. Therefore we consider FGF21 regulation as an adaptive response to hepatic steatosis. Improving FGF21 signaling in NAFLD patients enables novel treatment options. Conversely, repression of the hepatic glucose transporter GLUT2 implies an imbalance in glucose uptake and osmoregulation (Fig. 3 A1).

The combined lipid & TNFα treatment elicited strong induction of CXCL8, MAP3K8, MAP4K4, SOCS2 and SOCS3. Note, SOCS2 deletion was shown to protect against hepatic steatosis but worsens insulin resistance in high-fat-diet-fed mice [48] while ablation of SOCS3 enhances hepatic insulin sensitivity but increases lipogenesis resulting in fatty liver and obesity [49]. Furthermore, up-regulation of CXCL8 is of critical importance for the recruitment of neutrophils and was shown to cause tissue damage in NAFLD/NASH [50]. Inhibiting CXCL8 or blocking its receptor CXCR1 may proof to be effective in the treatment of NASH [51]. The up-regulation of MAP3K8 and MAP4K4 highlights responsiveness of the in-vitro system to cytokines. Pharmacological inhibition of MAP3K8 was reported to block human cytotoxic T lymphocyte effector functions [52] and may be therapeutically explored in the treatment of NASH. Likewise MAP4K4 inhibitors are explored in different disease indications linked to inflammation and cancer. Inducible deletion of Map4k4 in obese mice improved insulin sensitivity in liver and adipose tissue [53]. Further examples include repressed dystonin (DST) coding for a cytoskeletal protein and induced ACSL4, an enzyme that regulates fatty acid synthesis and lipid degradation (Fig. 3 B2).

We considered transcript changes of HepG2 cultures after daily lipid treatment for 7 days and observed sustained induction of CPT1α, CYP24A1 and PDK4 (Fig. 3 A3 and B3). The combined lipid and TNFα treatment elicited 13 common gene regulations. Transcriptional regulation of ACSL1, PDK4, MTTP, NR1H4 and PKLR highlight altered lipid and glucose metabolism while DYNC1L1, CYP24A1, CYP26A1 and HSDL2 point to ER/mitochondrial stress and LD growth (Fig. 3d). We also considered the time dependent changes in LD associated gene regulations in cultures of primary human hepatocytes (Fig. 3e). Two genes (ANGPTL4 and CPT1α) were expressed at any given time point while the gene coding for the cell death-inducing DFFA-like effector C was significantly induced at 24 h and 48 h; expression of PLIN2 and HSDL2 was prominent at 48 h and 72 h (Fig. 3e). Specifically, CIDEC is an LD associated protein to support LD growth by transferring neutral lipids from smaller to larger LDs and may interact with the perilipin 2 as observed by confocal immunofluorescence microscopy in co-localization studies (see below).

### Lipid-laden hepatocytes/NAFLD patient liver biopsy comparison

We recently reported the genomics of fatty liver disease in human NAFLD [54], and the data were compared to findings obtained from lipid-laden hepatocytes. Figure 3c depicts a Venn diagram and reveals the kinase PDK4 to be commonly regulated among all experimental conditions once again highlighting the importance of PDK4 in inhibiting the pyruvate dehydrogenase complex in hepatic steatosis. Additionally, DEGs regulated alike among the different experimental data sets were selected for protein-protein interaction network analysis. Here, a total of 108 statistically significantly regulated genes were considered (Additional file 6: Table S3) and are shown to participate in 155 protein-protein interactions. Therefore, evidence was obtained for mechanistically linked and lipid droplet associated genes to interact with each other when the coded proteins were considered (String network analysis; Fig. 3c).

### Immunofluorescence microscopy

**Fig. 4:**
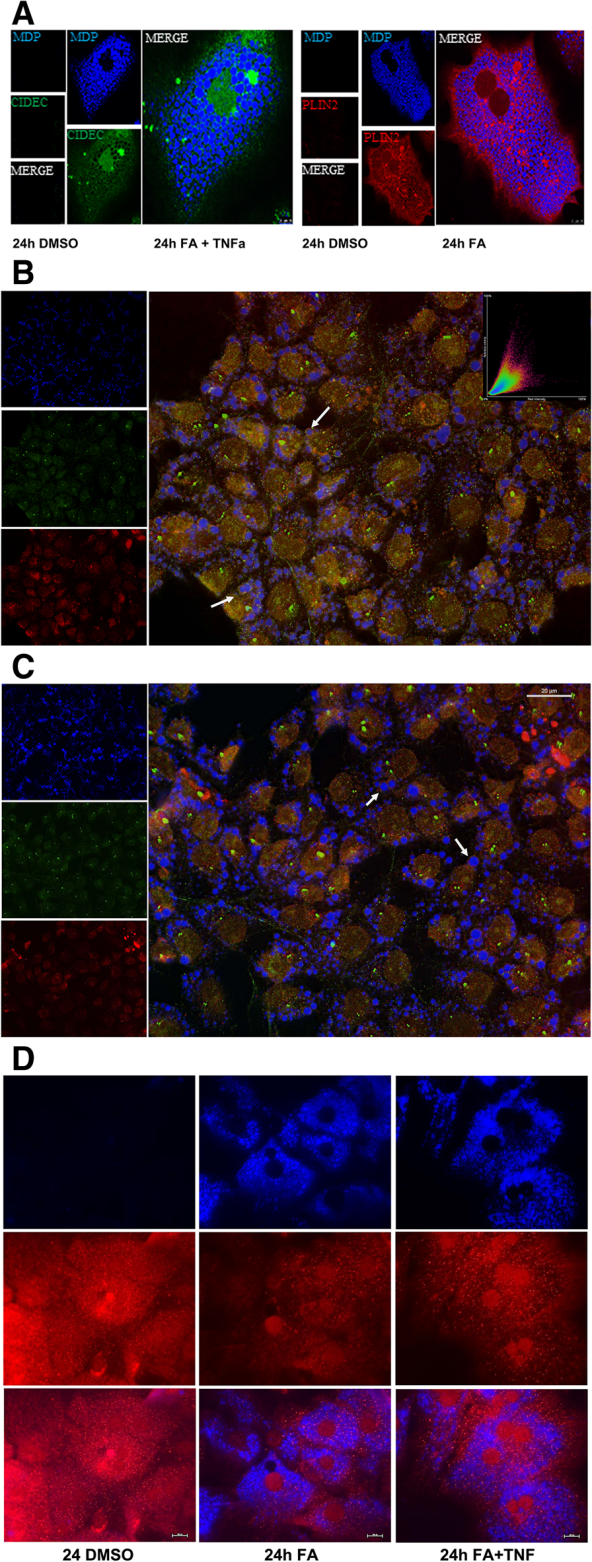
Immunofluorescence imaging of PLIN2, CIDEC and CPT1A protein expression in steatotic primary human hepatocyte cultures. **a** Primary human hepatocyte cultures were treated with a mixture of 0.5 mM PA/OA and stained individually for PLIN2 or CIDEC. Lipids were stained with the blue fluorescent dye MDP. CIDEC is given in green; PLIN2 expression is shown in red. **b** Co-localization of PLIN2 and CIDEC on the surface of LD monolayers. Primary human hepatocyte cultures were treated with a mixture of 0.5 mM PA/OA for 24 h and lipids were visualized the blue fluorescent dye MDP. Immunofluorescent staining of PLIN2 and CIDEC is given in the red and green channel, respectively. The co-localization image analysis was computed with a Pearson’s correlation coefficient of 0,806,395, a Manders overlap = 0,942,398 with K1; K2 = 0,751,118; 1,182,389 and a co-localization coefficient = 1:1. A scatter blot of the co-localization fluorescent signals is given as an insert. **c** Co-localization of PLIN2 and CIDEC on the surface of LD monolayers. Primary human hepatocyte cultures were treated with a mixture of 0.5 mM PA/OA and TNFα for 24 h and lipids were visualized the blue fluorescent dye MDP. Immunofluorescent staining of PLIN2 and CIDEC is given in the red and green channel, respectively. The bar represents 20 μm. **d** Immunofluorescence imaging of carnitine palmitoyltransferase 1A (CPT). Primary human hepatocyte cultures were treated with a mixture of 0.5 mM PA/OA for 24 h. Lipids were stained with the blue fluorescent dye MDP and CPT1A is shown in red. The images were captured with the Nikon TiE phase contrast immunofluorescent microscope with a 60x objective using the NIS elements software (version 4.13.); the scale bar is given in μm. The merged channels are given as large images and single channels are shown as smaller images

**Fig. 5:**
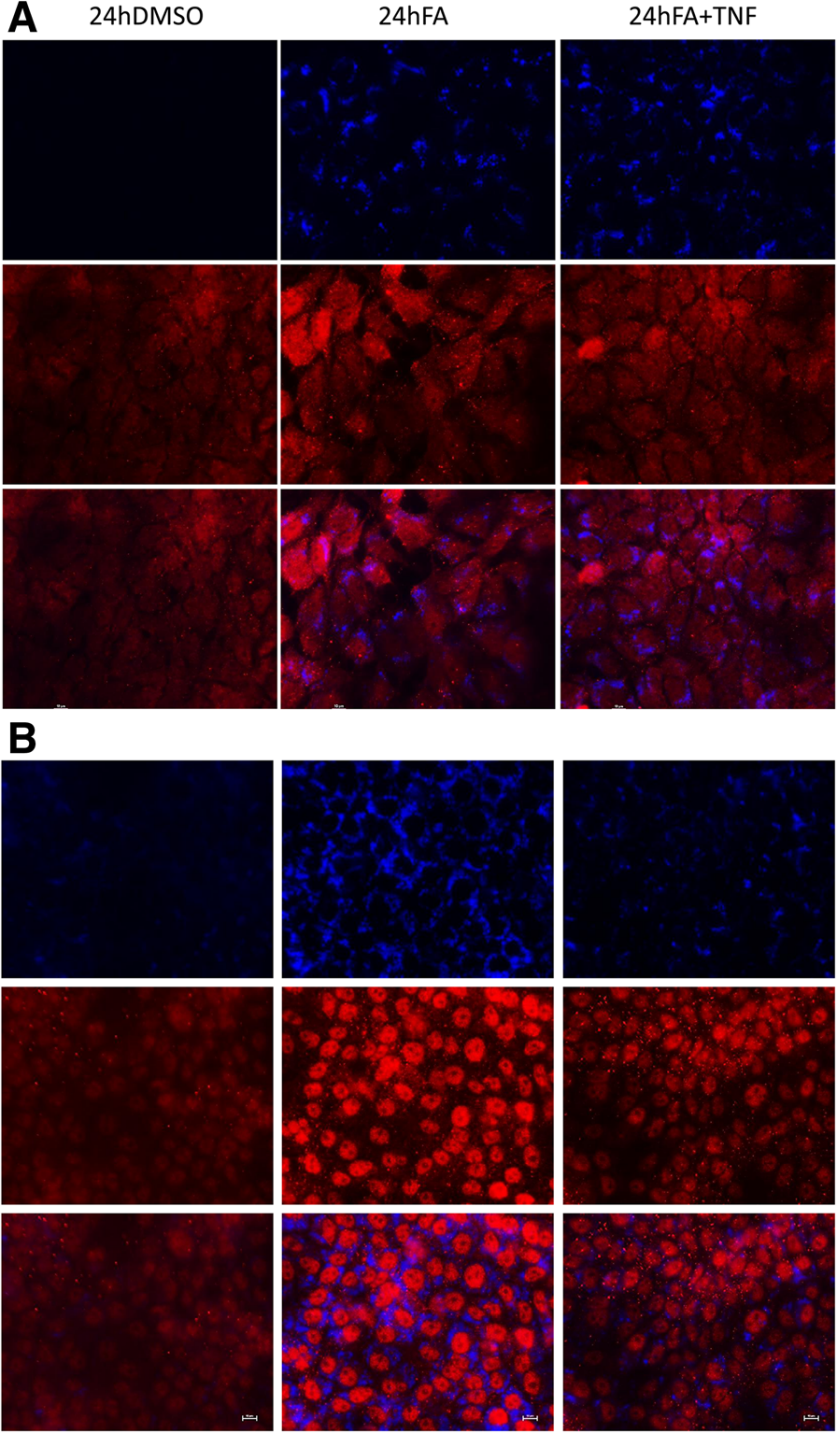
Immunofluorescence imaging of HILPDA and ROCK2 in Huh7 cells. Cultures of the human hepatoma cell line Huh7 were treated with a mixture of 0.5 mM PA/OA or the combined lipid and TNFα treatment and stained individually for HILPDA or ROCK2. Lipids were stained with the blue fluorescent dye MDP. **a** HILPDA (red) and lipid droplets (blue). Shown in the upper panel is the lipid stain; in the second panel HILPDA expression is given and the lower panel depicts the merged images. **b** ROCK2 (red) and lipid droplets (blue). Shown in the upper panel is the lipid stain; in the second panel ROCK2 expression is given and the lower panel depicts the merged images. The images were captured with Nikon TiE inverted microscope (images were captured with a 60x objective and the scale bar is given in μm)

We selected the targets CIDEC, CPT1A, HILPDA, HIF1α, JAK1, PDK4, PLIN2 and ROCK2 to further evaluate expression of the protein and therefore druggability by immunofluorescence imaging. Additionally, multicolor imaging studies were performed to probe for co-localization of CIDEC and PLIN2 on lipid droplets (Figs. 4, 5). Shown in Fig. 4 panel A is the immunofluorescence imaging of CIDEC (green channel) and PLIN2 (red channel). The lipid droplets are stained with the blue fluorescent dye monodansylpentane. Note the marked expression of CIDEC and PLIN2 in lipid-laden primary human hepatocyte cultures. Shown in panel B is the co-localization of CIDEC and PLIN2 on lipid droplets in proximity to each other. A similar picture emerged after the combined lipid and TNFα treatment of PHHs (Fig. 4, panel C) and both proteins dress the LD monolayer in proximity. Conversely, CPT1A is markedly reduced in steatotic hepatocyte cultures, and a similar reduction in CPT1A protein was observed after the combined lipid and TNFα treatment of PHHs (Fig. 4, panel D). It is tempting to speculate that the reduction in CPT1A protects mitochondria against lipid overload. Notwithstanding, CPT1A mRNA was induced in expression (Fig. 3, panel A1-A3) in PHHs and hepatoma cell lines after lipid but not the combined lipid and TNFα treatment.

We next considered the expression of HILPDA and Rock2 (Fig. 5). Although insignificantly changed at the mRNA level both targets are of critical importance in LD biogenesis (HILPDA) and actin cytoskeleton dynamics (ROCK2). Note, the combined lipid and TNFα treatment of HuH7 cells significantly repressed dystonin mRNA (see Fig. 3b) and this protein functions as an intermediate filament of the cytoskeleton. Immunofluorescence microscopy revealed marked expression of HILPDA (panel A) and the kinase ROCK2 (panel B) in lipid treated Huh7 cell cultures; however the combined lipid and TNFα treatment diminished their expression.

**Fig. 6:**
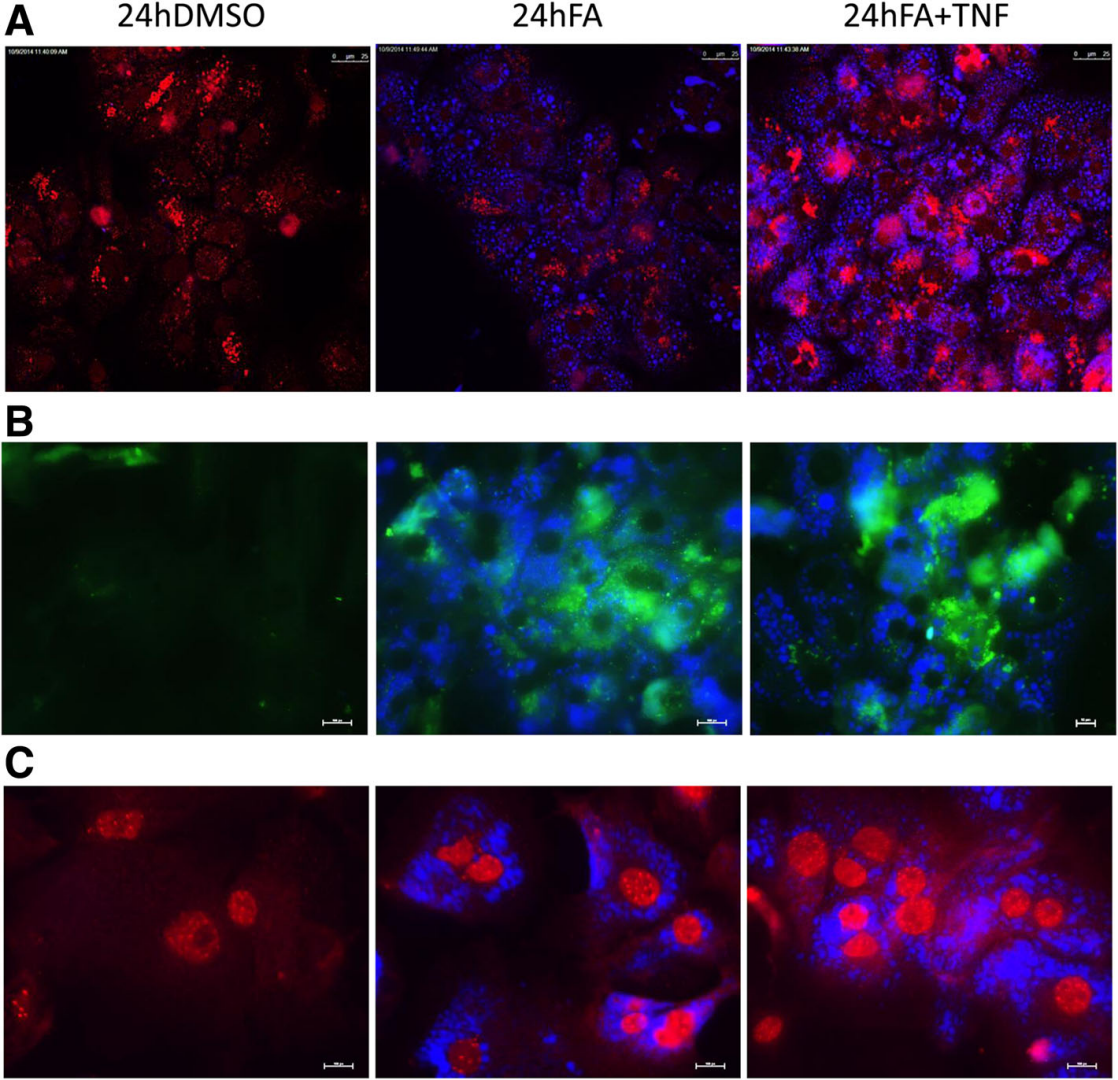
Immunofluorescence imaging of Hif1, PDK4 and JAK1 in primary human hepatocyte cultures. Cultures of primary human hepatocytes were treated with a mixture of 0.5 mM PA/OA or the combined lipid and TNFα treatment and stained individually for HIF1α, PDK4 or JAK1. Lipids were stained with the blue fluorescent dye MDP. Depicted are merged images. **a** HIF1α (red) and lipid droplet (blue). **b** PDK4 (green) and lipid droplets (blue). **c** JAK1 (red) and lipid droplets (blue). The images were captured with Nikon TiE inverted microscope (images were captured with a 60x objective and the scale bar is given in μm)

We also investigated regulation of HIF1α, JAK1 and PDK4 by immunofluorescence microscopy in cultures of primary human hepatocytes (Fig. 6, panels a-c) and observed induced expression of the proteins in lipid-laden hepatocytes particularly by the combined lipid and TNFα treatment.

**Fig. 7:**
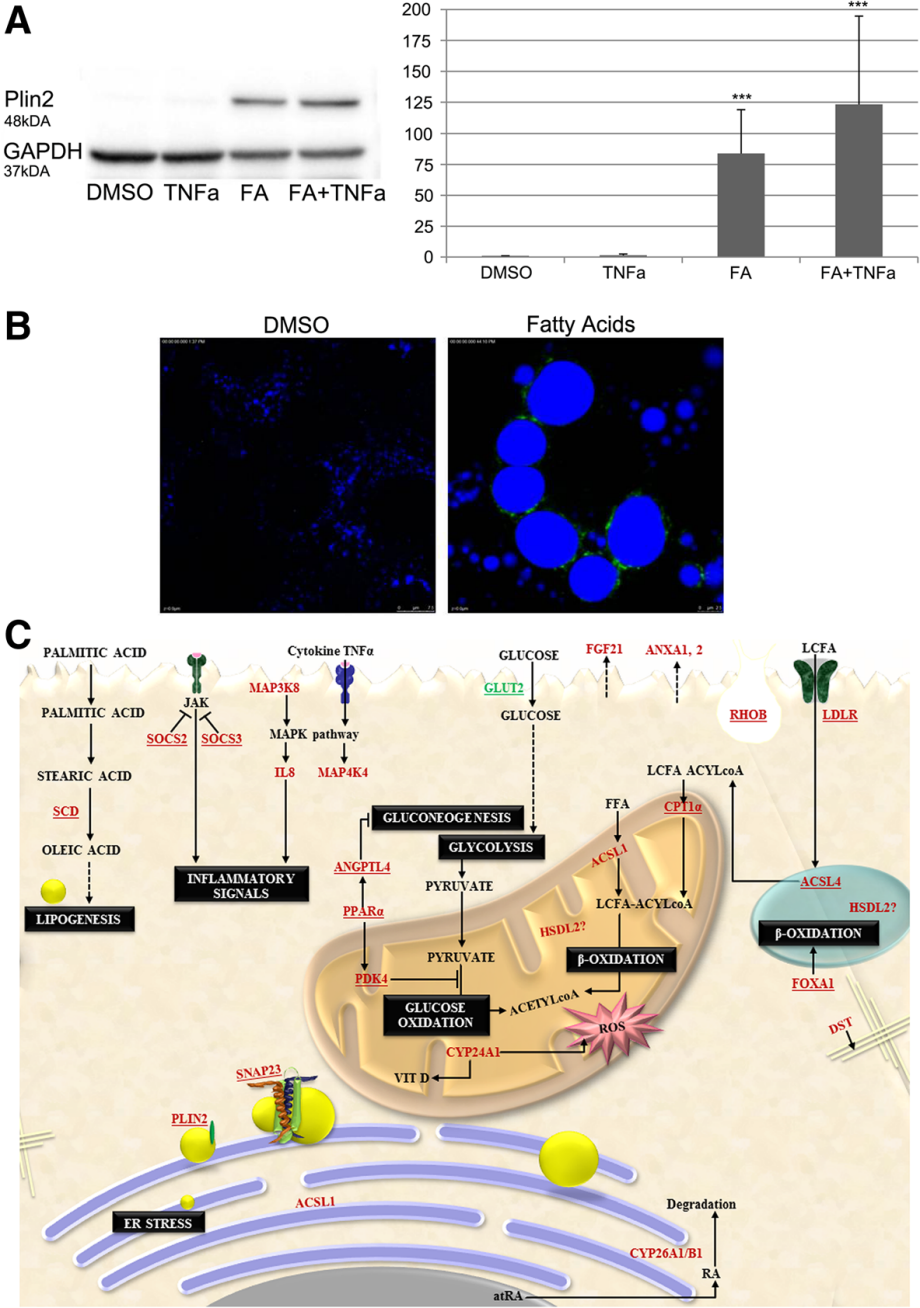
Western blotting and immunofluorescence imaging of PLIN2 and a schematic overview of LD associated gene regulations in lipid-laden hepatocytes. Cultures of primary human hepatocyte and hepatoma cell lines were treated with a mixture of 0.5 mM PA/OA or the combined lipid and TNFα treatment. **a** Western blotting of Plin2 with GAPDH as loading control.*** *p* < 0.01. **b** Immunofluorescence of DMSO vehicle and lipid treated hepatocytes highlighting PLIN2 localization around the monolayer of LDs. The lipids were stained with MDP dye and the scale bar is 7.5 μm. **c** Overview of mechanistically linked and LD associated genes in an in-vitro model of fatty liver disease. Depicted is a summary of NAFLD responsive genes in steatotic hepatocyte cultures. Fatty acid uptake occurs either by diffusion or lipid transporters. Continuous supply of lipids leads to an imbalance in glycolysis and gluconeogenesis that in turn affects β-oxidation of fatty acids. The excess lipids are stored in the form of lipid droplets. Simultaneously, genes responsible for LD growth are up-regulated and support LD expansion. The hepatocytes also respond to external stimulus such as TNFα signals by up-regulating mitogen activated protein kinases as well as other inflammatory signals such as interleukins and suppressors of cytokine signaling. Overall, cellular homeostasis is disturbed leading to enhanced lipid synthesis and altered metabolic signaling and eventually augments lipotoxicity. Figure 7c had been created by the authors

Additionally, we performed WB experiments (Fig. 7, panel A) in hepatocyte cultures and found PLIN2 to be strongly induced by about 77 and 124-fold after lipid and the combined lipid & TNFα treatment. Note the distinct association of PLIN2 with the monolayer of the lipid droplets shown in Fig. 7 panel B. Lastly, a summary of major findings is given in Fig. 7c.

## Discussion

Fatty liver disease has become a major health burden in Western societies and there is unmet need for new medication. To facilitate the drug discovery process and to search for new drug targets a gene expression profiling study was performed. Based on our recent review on the molecular pathophysiology of lipid droplet formation in hepatocytes the genomic data were filtered for approximately 300 genes mechanistically linked to lipid transport, lipogenesis, LD growth, glucose and fatty acid metabolism as well as signaling events [4]. Besides, lipid growth over time was studied, and the morphological changes of lipid-laden hepatocyte cultures mimic, at least in part, changes observed in-vivo. We also evaluated inhibitors of FATP2 and dynamin in lowering hepatic intracellular lipid concentrations. Altogether, LD-associated gene regulations and perturbed metabolic pathways were identified, and LD associated proteins such as perilipin 2 were confirmed by immunofluorescence microscopy dressing the LD monolayer. Therefore, applying genomics to lipid-laden hepatocytes helped to pinpoint putative drug targets and to ascertain adaptive responses to hepatic steatosis. Due to the fact that primary human hepatocyte cell cultures are not routinely available the genomics of lipid-laden human hepatoma cells was also compared to findings obtained with PHH. Furthermore, we investigated the effects of TNFα treatment to mimic inflammation as observed in steatohepatitis. Altogether known and putative drug targets were identified including the LD-growth and LD-fusion associated PLIN2, PLIN3, SNAP23 VAMP and the kinases PDK4, JAK, MAPK and ROCK2. Moreover, clinical relevance was established by comparing the genomic data from in-vitro studies with findings obtained from NAFLD patient biopsies.

### Induction of hepatic steatosis and lipid droplet growth

Initially, two approaches were employed to induce steatosis either by treatment with DTT, a reducing agent to cause ER stress and ectopic LD budding or by treatment of hepatocyte cultures with a mixture of palmitic and oleic acid. Both treatments resulted in lipid droplet formation (Fig. 1). However, DTT even at lower concentrations was highly toxic and induced smaller sized LDs whereas PA/OA treatment was well tolerated and enabled an assessment of LD fusion by live cell imaging. Importantly, over-nutrition, lack of satiety and the resultant obesity can lead to fatty liver disease while consumption of a high-fat diet rapidly exacerbates the development of NAFLD especially at elevated glucocorticoid levels [55]. In order to mimic a high-fat “Western diet” the PA/OA treatment was considered in detail and the intracellular lipid content was quantified over time (Fig. 2). Specifically, one hour after fatty acid treatment the intracellular lipid content was significantly increased (Fig. 2 and Additional file 1: Figure S1). Apparently the hepatic lipid uptake followed two phases, i.e. the period 4 h to 24 h with a gradual increase and 24 h to 48 h where a steep increase but subsequent decline was observed at 72 h. Supplementing the culture media with the LD fusogen propranolol accelerated LD growth and fusion (Additional file 3: Figure S3B) and this ß-adrenoceptor antagonist worsens liver injury in a model of non-alcoholic steatohepatitis [29].

The morphological features of steatosis and steatohepatitis include ballooning of hepatocytes; stressed organelles affect ER, mitochondria and peroxisome functioning [4, 56]. Additionally, the large number of intracellular LD influences cytoskeletal dynamics and signaling events. We observed on average 50 lipid droplets per cell after treatment with PA/OA for 24 h. After 48 h and 72 h of treatment lipid droplets grew in size but declined in number. This was accompanied with an increase in hepatocyte surface area (data not shown). Collectively, the morphological changes of lipid-laden hepatocyte cultures mimic, at least in part, changes observed in-vivo*.* Moreover, the LD growth and fusion associated gene regulations (PLIN2, PLIN3, SNAP23, VAMP etc.) provide evidence for the relevance of the in-vitro model to recapitulate changes observed in-vivo.

### In-vitro hepatic steatosis response genes

The genomic responses to lipid treatment were investigated in PHH and hepatoma cell lines. Note, hepatoma cell lines are easy to use and for a given cell line data reproducibility is robust. Conversely, primary human hepatocytes (PHH) are difficult to obtain, costly and next to donor dependent responses PHHs can only be cultured for a short period of time. Consequently, there are important tradeoffs that need to be considered when comparing the different in-vitro systems. Although genomic responses to steatosis differed among the in-vitro systems commonalities were observed when the data were filtered for genes mechanistically linked to lipid droplet formation and metabolism (Additional file 5: Table S2). Hydroxysteroid dehydrogenase like 2 was commonly regulated among all systems, and the protein functions as desaturase in mitochondria and peroxisomes [37].

A comparison of in-vitro genomic data and human NAFLD biopsy findings (Fig. 3c) revealed the mitochondrial pyruvate dehydrogenase kinase 4 (PDK4) as commonly up-regulated. This kinase is of key importance in glucose metabolism. Expression of PDK4 is influenced by HIF1α and retinoic acid and the retinoic acid hydroxylase CYP26A was significantly up-regulated in lipid-laden hepatocyte cultures. Previous research demonstrated CYP26A1 and CYP26B1 to be induced in expression by all-trans retinoic acid [57–59]. Furthermore, CYP monooxygenases catalyze oxidization of fatty acids. The induced expression of PPARα in HepG2 cells and PPARβ/δ in primary human hepatocyte cultures stimulates PDK4 activity which in turn inhibits the conversion of pyruvate to acetyl-CoA. Next to PDK4 other target of PPARβ/δ such as ANGPTL4 and PLIN2 were significantly up-regulated. Induction of perilipin 2 is typically augmented in LD biogenesis as observed in cultures of primary human hepatocytes and the hepatoma cell lines (Fig. 4). Conversely, the glucose transporter 2 was significantly repressed in expression indicating an imbalance in glucose uptake and osmoregulation. The protein facilitates bidirectional glucose transport, and the complex interactions between glucose and lipid metabolism in fatty liver disease have been the subject of several reviews [4, 60]. Interestingly, Exendin-4, i.e. a glucagon-like peptide-1 receptor agonist improved fatty liver disease in ob/ob mice by regulating glucose transporter expression [61]; alike SREBP1c mediates glucose-stimulated GLUT2 gene expression in hepatocytes [62]. With cultures of primary human hepatocytes significant induction of angiopoietin-like 4 was observed that can be explained by the combined activity of PPARβ/δ and HIF1α [42]. Angiopoietin-like 4 was also shown to be a direct target of glucocorticoid receptor and participates in glucocorticoid-regulated triglyceride metabolism [43]. Induction of the growth factor FGF21 in PHH cultures is of great importance. This growth factor is predominantly secreted from the liver and ameliorates fatty liver disease through activation of the AMPK-SIRT1 pathway [63]. Another NAFLD regulated gene is carnitine palmitoyltransferase 1A. The coded protein is part of the carnitine shuttle in transferring long chain fatty acids to the inner mitochondrial membrane to initiate the process of fatty acid ß-oxidation. Unlike induced expression of CPT1A transcripts the protein was repressed (Fig. 4d) possible to protect mitochondria from lipid overload.

Figure 7 depicts a summary of the study findings highlighting the following results:

Steroyl coA desaturase is induced in lipid-laden hepatocyte cultures. The ER localized enzyme catalyzes the δ9 desaturation of fatty acyl-CoA substrates including palmitoyl- and stearoyl-CoA [38]. It is a key enzyme for the biosynthesis of monounsaturated fatty acids which are essential building blocks for the synthesis of hepatic TG and cholesterol esters and are exported via the VLDL secretory pathway. During LD growth PLIN2 expression is augmented and a recent study evidenced Plin2 to inhibit cellular glucose uptake through interactions with SNAP23, a SNARE complex protein [64]. Specifically, LD fusion is supported by the SNARE complex and SNAP23 is part of it. This vesical-membrane fusion protein was identified as frequently up-regulated in our genomics study of human NAFLD [54]; its common regulation signifies the importance of SNARE proteins in facilitating lipid droplet fusion with implication on glucose uptake and insulin sensitivity as suggested by Boström and colleagues [65].

During an initial phase mitochondrial ß-oxidation of fatty acids is increased in hepatic steatosis. Testimony to an altered mitochondrial lipid metabolism is the up-regulation of CPT1α mRNA, i.e. a component of the carnitine acyltransferase system. However, the CPT1A protein was reduced in expression possibly to protect mitochondria from excessive lipid metabolism. Additionally, the peroxisomal acyl-CoA synthetase ACSL4 catalyzes activation of arachidonic and eicosapentaenoic acid [66]; its up-regulation influences synthesis of prostaglandins but also peroxisomal ß-oxidation of long chain fatty acids to shorter ones while increased expression of ACSL1 and CPT1α supports fatty acid metabolism in mitochondria. A recent report suggests overexpression of ACSL1 to correct mitochondrial dysfunction with increased coupling efficiency and decreased proton leakage [67]. Moreover, CPT1α as well as ACSL1/ACSL4 may modulate activity of transcription factors by influencing intracellular availability of fatty acids and acyl-CoA derivatives which are ligands for various transcription factors including hepatic nuclear factor 4α and PPARs [68, 69]. The present study revealed up-regulation of PPARβ/δ and well known target genes of this transcription factor, i.e. PDK4, ANGPTL4 and PLIN2.

To mimic the condition of inflammation hepatocyte cell cultures were treated with lipids and TNFα. Among the different systems the human hepatoma cell line HuH7 was most responsive with significant up-regulation of the genes coding for SOCS3, CXCL8 and mitogen activated protein kinases. Note, liver-specific suppressor of cytokine signaling-3 deletion in mice enhanced hepatic insulin sensitivity but increased lipogenesis to result in fatty liver disease and obesity [49]. Alike, CXCL8 was significantly increased in NASH patients as compared to bland steatosis or healthy controls [70] thus demonstrating clinical relevance of the current findings with hepatocyte cultures. Furthermore, induction of FOXA1 is an adaptive response to hepatic steatosis and was shown to reduce lipid accumulation in human hepatocytes [39] as is the induction of FGF21 with its great therapeutic potential in the treatment of NAFLD [40].

### Pharmacological inhibition of lipid uptake

Next to passive diffusion fatty acid uptake is dependent on transporters/translocases as well as receptor mediated mechanisms. Additionally, hepatic lipid uptake may involve lipid-rafts, calveolin- and clathrin mediated endocytosis. We therefore investigated the effects of FATP and dynamin inhibitors on hepatic lipid uptake by treating hepatocyte cultures with the small molecules CB16.2 and dynasore. Importantly, the fatty acid transporter FATP2 is expressed in the liver, and this protein is unique in a sense as it additionally functions as an acyl-CoA synthetase to support bile acid synthesis [71]. FATP2 augments uptake of long-chain fatty acids (LCFAs); therefore it regulates intracellular fatty acid pool size in hepatocytes [72]. In response to FATP2 inhibition lipid uptake in HepG2 cells was significantly reduced by 35% (see Fig. 2b) and a similar 30% reduction in hepatic lipid uptake was observed with dynasore (Fig. 2a). Interestingly, dynasore was found in a screen of ~ 16,000 compounds aimed at identifying inhibitors of GST-Grb2-stimulated dynamin2 [34], and this GTPase is of critical importance in endocytosis and intracellular cholesterol transport [33, 73]. Our study confirms fatty acid uptake to involve fatty acid transport proteins and lipid mediated endocytosis. The in-vitro systems can therefore be used to investigate experimental inhibitors effectively.

### Genomic responses to hepatic steatosis reveal bona fide drug targets

The genomic studies revealed a range of rational and putative drug targets. Specifically, we observed up-regulation of the microsomal triglyceride transfer protein (MTTP). In 2012 Lomitapide was approved as the first in class MTTP inhibitor for the treatment of family hypercholesterolemia. The genomic study also revealed regulation of the nuclear receptors PPARα and PPARβ/δ in addition to CPT1. Importantly, clofibrate and fenofibrate activate the PPARα transcription factor and are well established lipid lowering agents with fenofibrate being used for decades in the clinic. Thus, the therapeutic benefit of this drug target was already demonstrated. Alike, the transcription factor PPAR β/δ is a key regulator of glucose and lipid metabolism, and different agonists have been developed to increase lipid metabolism and to improve insulin sensitivity as well as serum lipid profiles [74]. The present study revealed the PPAR β/δ targets ANGPTL4, PDK4 and HILPDA as highly interesting proteins for the development of inhibitors. Recently, a PDK2 inhibitor targeting the ATP binding pocket was reported to improve glucose tolerance and to reduce hepatic steatosis by affecting pyruvate dehydrogenase complex activity [75]. Research also identified HILPDA to inhibit adipose triglyceride lipase thus providing a rationale to inhibit activity of this protein in NAFLD [76]. Furthermore, topical inhibitors of PPAR β/δ are developed to treat psoriasis [77]. Conversely, Etomoxir is an irreversible inhibitor of CPT1 and blocks fatty acid uptake and fatty acid ß-oxidation in mitochondria. This drug was evaluated in patients with heart failure and type 2 diabetes; however, clinical trials were terminated due to severe hepatotoxicity. Treatment of primary human hepatocyte cultures with lipids caused a marked reduction in CPT1A protein as evidenced by immunofluorescence (Fig. 4d). Note, a recent study demonstrated recovery of CPT1A to ameliorated hepatic steatosis in mice after treatment with the hepatic stimulator substance (HSS). A similar result was obtained when steatotic HepG2 cells were transfected with a plasmid for HSS and the effect was enhanced by C75, a CPT-1 activator [78]. Moreover, increased hepatic mitochondrial fatty acid oxidation reversed insulin resistance and glucose intolerance in obese mice independently of hepatic steatosis [79].

Additional file 5: Table S2 informs on NAFLD responsive genes which are regulated in common in in-vitro*/*in-vivo comparisons. Several of the coded proteins are bona fide therapeutic targets and include the FOXO proteins. The FOXO1 transcription factor plays a decisive role in glucose and lipid metabolism. Inhibition of its activity by AS1842856 effectively lowers lipogenesis. The observed regulation of AKT and FOXO3 is also suggestive for mTORC1-independent regulation of autophagy as summarized by Ward et al. [80]. FOXO inhibitors are therefore a hot topic in development of new drugs for the treatment of metabolic disorders [81, 82].

The present study identified induced PDK4 in response to hepatic steatosis and this kinase functions as a metabolic switch between glucose and fatty acid metabolism. Independent studies revealed PDK4 activity to be influenced by thiazolidinediones and pioglitazone was shown to improve liver histology and fibrosis in patients with non-alcoholic steatohepatitis [83].

Lipid droplet associated proteins and their inhibition have become the focus of recent research [84] and the present study evidences exclusive expression of perilipin 2 and 3 in hepatic steatosis with PLIN2 being commonly regulated in-vitro and in-vivo (Additional file 5: Table S2). Importantly, rational drug design based on 3D crystallography revealed 4-Nitrophenyl 2,3,4-Tri-O-levulinoyl-Î ± −D-mannopyranoside as an inhibitor for perilipin 1 [85] while perilipin 2 inhibition is considered to be a novel strategy in treating NAFLD and other age-related diseases [86].

Consistently, we observed regulation of flotilin-1; this lipid-raft associated protein is of critical importance in the budding of LDs and their growth. Given that endocytosis of flotillin-1 and flotillin-2 is regulated by Fyn kinase [87] its pharmacological inhibition may proof to be beneficial in treating progressive disease and particularly NASH. Moreover, among commonly regulated hepatic steatosis genes is FGF21. This growth factor belongs to a group of signaling proteins and was shown to ameliorate the condition of NAFLD. In a recent review the application of FGF21 analogues for the treatment of metabolic disorders and NAFLD has been summarized [88, 89].

Up-regulation of the LDL receptor in response to lipid treatment was another important finding and inhibition of LDLR internalization by dynasore was effective in lowering the intracellular lipid content as demonstrated in the present study and as reviewed by Preta et al. [90]. There are also reports demonstrating beneficial effects of L- and N-type calcium channel blocker on glucose and lipid metabolism in patients with hypertension and Type II diabetes [91].

These and other examples demonstrate the utility of the genomic approach in identifying novel drug targets for the treatment of fatty liver disease.

### Study limitations

Although some key aspects of hepatic steatosis such as lipid droplet formation can be investigated in cultures of human hepatocytes the following caveats need to be considered: In-vivo progressive fat accumulation of the liver evolve through different mechanisms, i.e. over-nutrition, drug induced, ER stress etc. and eventually stimulates the release of pro-inflammatory chemokines either by stressed adipocytes or injured hepatocytes which cannot be precisely mimicked in a cell culture system. Additionally, TNFα treatment of lipid-laden hepatocytes mimics inflammation with TNFα serum levels being elevated in NASH patients. However, in-vivo the immune response augmented by Kupffer cells perpetuates acinar/lobular inflammation and involves mobilization and infiltration of neutrophils into the sinusoidal space to contribute to disease progression which cannot be studied in-vitro. Moreover, intrahepatic CD8 and natural killer T cells aggravate the condition of NASH and cytokine stress with alarm signals sent by harmed hepatocytes to stimulate Toll like receptor signaling and an immune response. The complex interactions between endothelial cells of the sinusoids and hepatic stellate cells, their activation to myofibroblasts within the space of Disse and additional interactions with immune cells likely enhance deposition of extracellular matrix components and secretion of inhibitors of matrix metalloproteinases cannot be studied in in-vitro systems and therefore represent a significant limitation, particularly when biological processes associated with progressive liver disease are investigated.

## Conclusions

We report genomic responses of primary human hepatocyte and hepatoma cell cultures to the condition of steatosis. We observed regulation of genes mechanistically linked to lipid droplet formation and NAFLD associated changes in mitochondrial lipid and glucose metabolism. Our research identified LD associated putative drug targets and among the most promising candidates are the CIDE and perilipin family of proteins. Likewise, proteins of the SNARE complex, i.e. SNAP23 and the vesicle-associated membrane protein 3 as well as the endoplasmic reticulum lipid raft-associated protein 1 are bona fide targets. Collectively, lipid droplet associated proteins can be explored for therapeutic intervention strategies, and the cell culture models proofs to be valuable for an identification of putative targets and an evaluation of drug candidates for the treatment of NAFLD.

## Additional files

Additional file 1: **Table S1.** Patient characteristics of hepatocyte donors. (PDF 170 kb)

Additional file 2: **Figure S1.** Quantification of intracellular lipid content over time. **(A)** The plotted graph depicts three treatment conditions, i.e. HepG2 cells without any treatment (K-) or after treatment with the DMSO vehicle control (K+) or after treatment with a 1:1 mixture of the fatty acids OA/PA for 1 h, 2 h, 4 h, 6 h, 24 h, 48 h and 72 h. After 6 h of treatment, the lipid content increased by 25 μg/ml (K+, *n* = 28; PA/OA, *n* = 23); after 24 h by 24 μg/ml (K+, *n* = 15; PA/OA, *n* = 18), after 48 h by 70 μg/ml (K+, *n* = 14; PA/OA, *n* = 17) and after 72 h by 71 μg/ml (K+, *n* = 7; PA/OA, n = 14) when compared to the DMSO vehicle control (K+), respectively. (T-Test K+ ↔ PA/OA: 1 h, *p* ≤ 0,00; 2 h, *p* ≤ 1,87 × 10^− 6^;4 h, *p* ≤ 3,5 × 10^− 7^; 6 h, *p* ≤ 6,3 × 10^− 10^; 24 h, *p* ≤ 2,4 × 10^− 7^; 48 h, p ≤ 2,6 × 10^− 11^; 72 h, *p* ≤ 1,6 × 10^− 11^). *corresponds to a *p*-value of *p* ≤ 0,001. **(B)** The histogram visualizes the increase in intracellular lipid content over time as compared to the DMSO vehicle control. (PDF 356 kb)

Additional file 3: **Figure S2.** Live cell imaging of LD fusion. Live cell images of HuH7 cells treated with 0.5 mM of 1:1 mixture of oleic acid and palmitic acid for 24 h. The smaller LDs (orange arrows) appear to fuse with larger LDs (red arrows). Panel B represents the initiation of fusion events where two LDs (v = 0.86 μm^3^; v = 2.9 μm^3^) and LDs (v = 1.49 μm^3^; v = 6.88 μm^3^) fuse to form a larger LD (v = 3.2 μm^3^) and (v = 8.78 μm^3^), respectively (panel D). The figures were captured at 40x using time-lapse z-stack by phase contrast microscopy (Nikon TiE) and processed using the NIS elements software version 4.13. The lipid droplets were stained with the blue fluorescent MDP dye (images are not shown). The size bar refers to 10 μm. (PDF 192 kb)

Additional file 4: **Figure S3.** Live cell imaging of LD growth induced by fusogen. **(A)** Depicted are live cell images of HepG2 cells either **(A1)** without any treatment (K-) or **(A2)** treatment with the DMSO vehicle control. **(A3)** treatment with a 0.5 mM mixture of oleic acid and palmitic acid for 24 h; **(A4)** FA and 200 μM propranolol treatment for 3 h. The live cell images were captured at 40x using time-lapse z-stack by phase contrast fluorescence microscopy (Nikon TiE) and further analyzed using the NIS elements software version 4.13. **(B)** Depicted are live cell images of LD in HepG2 cells. Upon propranolol treatment the smaller LDs (orange arrows) appear to fuse with larger LDs (red arrows). The insets represent actively grown LDs. **(B1)** Clockwise: *r* = 9.14 μm, 12.79, 11.34, 7.9, 8.94, 9.04, 10.26, 11.38, 11.09, 10.28, 8.93. **(B2)** *r* = 12.41, 11.36, 12.46, 10.88; **(B3)** *r* = 16.33, 14.21, 11.11, 17.36; **(B4)** *r* = 19.25, 15.88, 19.54). **(B5)** The fusion events did not result in spherical lipid droplets; hence the volume could not be determined. The figures were captured at 40x using time-lapse phase contrast microscopy (Nikon TiE) and further processed using the NIS elements software version 4.13. The lipid droplets were stained with the blue fluorescent MDP dye (images are not shown). The size bar refers to 10 μm. (PDF 417 kb)

Additional file 5: **Table S2.** List of mechanistically linked steatotic genes. (PDF 185 kb)

Additional file 6: **Table S3.** Compilation of NAFLD responsive genes in in-vitro/in-vivo comparisons. (PDF 96 kb)

## Abbreviations

ACSL1/ACSL4: Acyl-CoA synthetase long-chain family member
AMPK: 5′ adenosine monophosphate-activated protein kinase
ANGPTL4: Angiopoietin-like 4
ANXA1/ANXA2: Annexin A
ARG1: Arginase 1
ATP: Adenosine triphosphate
CD8: Cluster of differentiation 8
CIDEC: Cell Death-Inducing DFFA-Like Effector C
CPT1α/CPT1A: Carnitine palmitoyltransferase
CXCL8: Chemokine (C-X-C Motif) Ligand 8
CYP24A1: Cytochrome P450, family 24, subfamily A, polypeptide 1
CYP26A1: Cytochrome P450, family 26, subfamily A, polypeptide 1
CYP26B1: Cytochrome P450, family 26, subfamily B, polypeptide 1
DAPI: 4′,6-Diamidin-2-phenylindol
DEG: Differentially expressed gene
DMEM: Dulbecco’s modified media
DMSO: Dimethyl sulfoxide
DTT: Dithiotriol
DYNC1L1: Light intermediate chains
ER: Endoplasmic reticulum
FA: Fatty acid
FATP2: Fatty acid transport protein 2
FDR: False discovery rate
FGF21: Fibroblast growth factor 21
FOXA1: Forkhead box A1
GLUT2: Glucose transporter 2
GRB2: Growth factor receptor-bound protein 2
GST: Glutathione-S-transferase
GTP: Hydrolyze guanosine triphosphate
HCC: Hepatocellular carcinoma
HIF1α: Hypoxia inducible factor 1, alpha
HSDL2: Hydroxysteroid dehydrogenase like 2
LAMB1: Laminin, Beta 1
LCFA: Long-chain fatty acid
LDLR: Low density lipoprotein receptor
MAP3K8: Mitogen-activated protein kinase 8
MAP4K4: Mitogen-activated protein kinase 4
MDP: Monodansyl pentane
MTTP: Microsomal triglyceride transfer protein
MUFA: Monounsaturated fatty acids
NR1H4: Nuclear receptor subfamily 1, group H, member 4
OA: Oleic acid
ORO: Oil red O
PA: Palmitic acid
PBS: Phosphate-buffered saline
PDK4: Pyruvate dehydrogenase kinase, isozyme 4
PKLR: Pyruvate kinase, liver and RBC (red blood cells)
PLIN2: Perilipin 2
PPAR: Peroxisome proliferator-activated receptor
RHOB: Ras homolog family member B
RMA: Robust multi-array average
ROS: Reactive oxygen species
SCD: Stearoyl-coA desaturase
SIRT1: Sirtuin1
SLC2A2: Solute carrier family 2
SNAP23: Synaptosomal-Associated Protein, 23 kDa
SNARE: Soluble NSF attachment protein receptor
SOCS2/SOCS3: Suppressor of cytokine signaling
SREBP1: Sterol regulatory element-binding protein 1
TG: Triglycerides
TNFα: Tumor necrosis factor alpha
VLDL: Very low density lipoprotein
