# Supplementary material for: Genomics of lipid-laden human hepatocyte cultures enables drug target screening for the treatment of non-alcoholic fatty liver disease
Source: BMC Med Genomics. 2018 Dec 14;11:111. doi: 10.1186/s12920-018-0438-7 (PMC6295111; doi:10.1186/s12920-018-0438-7)
Supplement: Supplementary file 1 — Table S1. Patient characteristics of hepatocyte donors. (PDF 170 kb) [file 12920_2018_438_MOESM1_ESM.pdf]

Supplementary Table S1: Patient characteristics of hepatocyte donors.

patient ID	gender	age	reason for liver surgery	medical history
donor 1	female	61-65	*colorectal* cancer *metastatic* to the *liver*	*BMI 27, non-smoking; no prior history of liver disease*
donor 2	female	50-55	*colorectal* cancer *metastatic* to the *liver*	*BMI 24, smoking cessation 10 years ago; no prior history of liver disease*
donor 3	female	56-60	*colorectal* cancer *metastatic* to the *liver*	*BMI 23, non-smoking; no prior history of liver disease*
donor 4	female	66-70	*colorectal* cancer *metastatic* to the *liver*	*BMI 27, non-smoking no prior history of liver disease*
donor 5	female	61-65	*colorectal* cancer *metastatic* to the *liver*	*BMI 30, non-smoking; no prior history of liver disease*
donor 6	male	71-75	*colorectal* cancer *metastatic* to the *liver*	*BMI 28, non-smoking; no prior history of liver disease*

None of the patients were clinically obese or required insulin therapy and had no prior history of liver disease. Except for one case the patients were never smokers. The age of the patients is given as a range to secure their anonymity.
