# Supplementary material for: Genomics of lipid-laden human hepatocyte cultures enables drug target screening for the treatment of non-alcoholic fatty liver disease
Source: BMC Med Genomics. 2018 Dec 14;11:111. doi: 10.1186/s12920-018-0438-7 (PMC6295111; doi:10.1186/s12920-018-0438-7)
Supplement: Supplementary file 2 — Figure S1. Quantification of intracellular lipid content over time. (A) The plotted graph depicts three treatment conditions, i.e. HepG2 cells without any treatment (K-) or after treatment with the DMSO vehicle control (K+) or after treatment with a 1:1 mixture of the fatty acids OA/PA for 1 h, 2 h, 4 h, 6 h, 24 h, 48 h and 72 h. After 6 h of treatment, the lipid content increased by 25 μg/ml (K+, n = 28; PA/OA, n = 23); after 24 h by 24 μg/ml (K+, n = 15; PA/OA, n = 18), after 48 h by 70 μg/ml (K+, n = 14; PA/OA, n = 17) and after 72 h by 71 μg/ml (K+, n = 7; PA/OA, n = 14) when compared to the DMSO vehicle control (K+), respectively. (T-Test K+ ↔ PA/OA: 1 h, p ≤ 0,00; 2 h, p ≤ 1,87 × 10− 6;4 h, p ≤ 3,5 × 10− 7; 6 h, p ≤ 6,3 × 10− 10; 24 h, p ≤ 2,4 × 10− 7; 48 h, p ≤ 2,6 × 10− 11; 72 h, p ≤ 1,6 × 10− 11). *corresponds to a p-value of p ≤ 0,001. (B) The histogram visualizes the increase in intracellular lipid content over time as compared to the DMSO vehicle control. (PDF 356 kb) [file 12920_2018_438_MOESM2_ESM.pdf]

Supplementary Figure S1: Quantification of intracellular lipid content over time

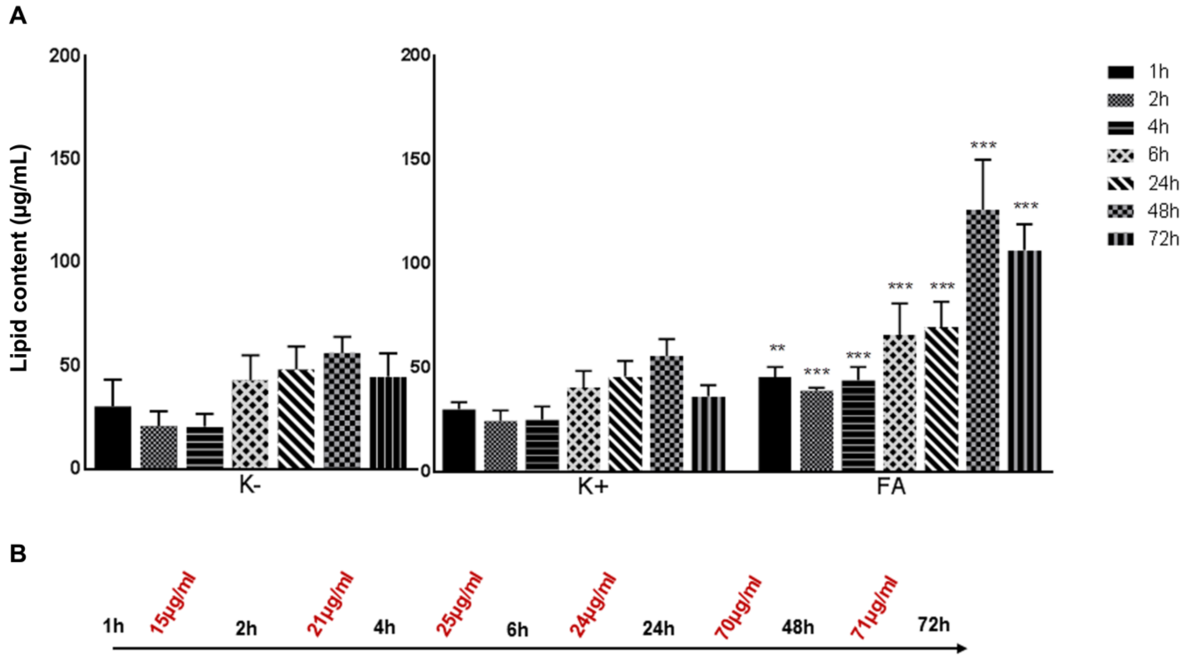

(A) The plotted graph depicts three treatment conditions, i.e. HepG2 cells without any treatment (K-) or after treatment with the DMSO vehicle control (K+) or after treatment with a 1:1 mixture of the fatty acids OA/PA for 1h, 2h, 4h, 6h, 24h, 48h and 72h. After 6h of treatment, the lipid content increased by 25µg/ml (K+, n=28; PA/OA, n=23); after 24h by 24µg/ml (K+, n=15; PA/OA, n=18), after 48h by 70µg/ml (K+, n=14; PA/OA, n=17) and after 72h by 71µg/ml (K+, n=7; PA/OA, n=14) when compared to the DMSO vehicle control (K+), respectively. (T-Test K+ ↔ PA/OA: 1h, $p \leq 0,00$; 2h, $p \leq 1,87 \times 10^{-6}$;4h, $p \leq 3,5 \times 10^{-7}$; 6h, $p \leq 6,3 \times 10^{-10}$; 24h, $p \leq 2,4 \times 10^{-7}$; 48h, $p \leq 2,6 \times 10^{-11}$; 72h, $p \leq 1,6 \times 10^{-11}$).

* corresponds to a p-value of $p \leq 0,001$.

(B) The histogram visualizes the increase in intracellular lipid content over time as compared to the DMSO vehicle control.
