# Supplementary material for: Genomics of lipid-laden human hepatocyte cultures enables drug target screening for the treatment of non-alcoholic fatty liver disease
Source: BMC Med Genomics. 2018 Dec 14;11:111. doi: 10.1186/s12920-018-0438-7 (PMC6295111; doi:10.1186/s12920-018-0438-7)
Supplement: Supplementary file 3 — Figure S2. Live cell imaging of LD fusion. Live cell images of HuH7 cells treated with 0.5 mM of 1:1 mixture of oleic acid and palmitic acid for 24 h. The smaller LDs (orange arrows) appear to fuse with larger LDs (red arrows). Panel B represents the initiation of fusion events where two LDs (v = 0.86 μm3; v = 2.9 μm3) and LDs (v = 1.49 μm3; v = 6.88 μm3) fuse to form a larger LD (v = 3.2 μm3) and (v = 8.78 μm3), respectively (panel D). The figures were captured at 40x using time-lapse z-stack by phase contrast microscopy (Nikon TiE) and processed using the NIS elements software version 4.13. The lipid droplets were stained with the blue fluorescent MDP dye (images are not shown). The size bar refers to 10 μm. (PDF 192 kb) [file 12920_2018_438_MOESM3_ESM.pdf]

Supplementary Figure S2: Live cell imaging of LD fusion.

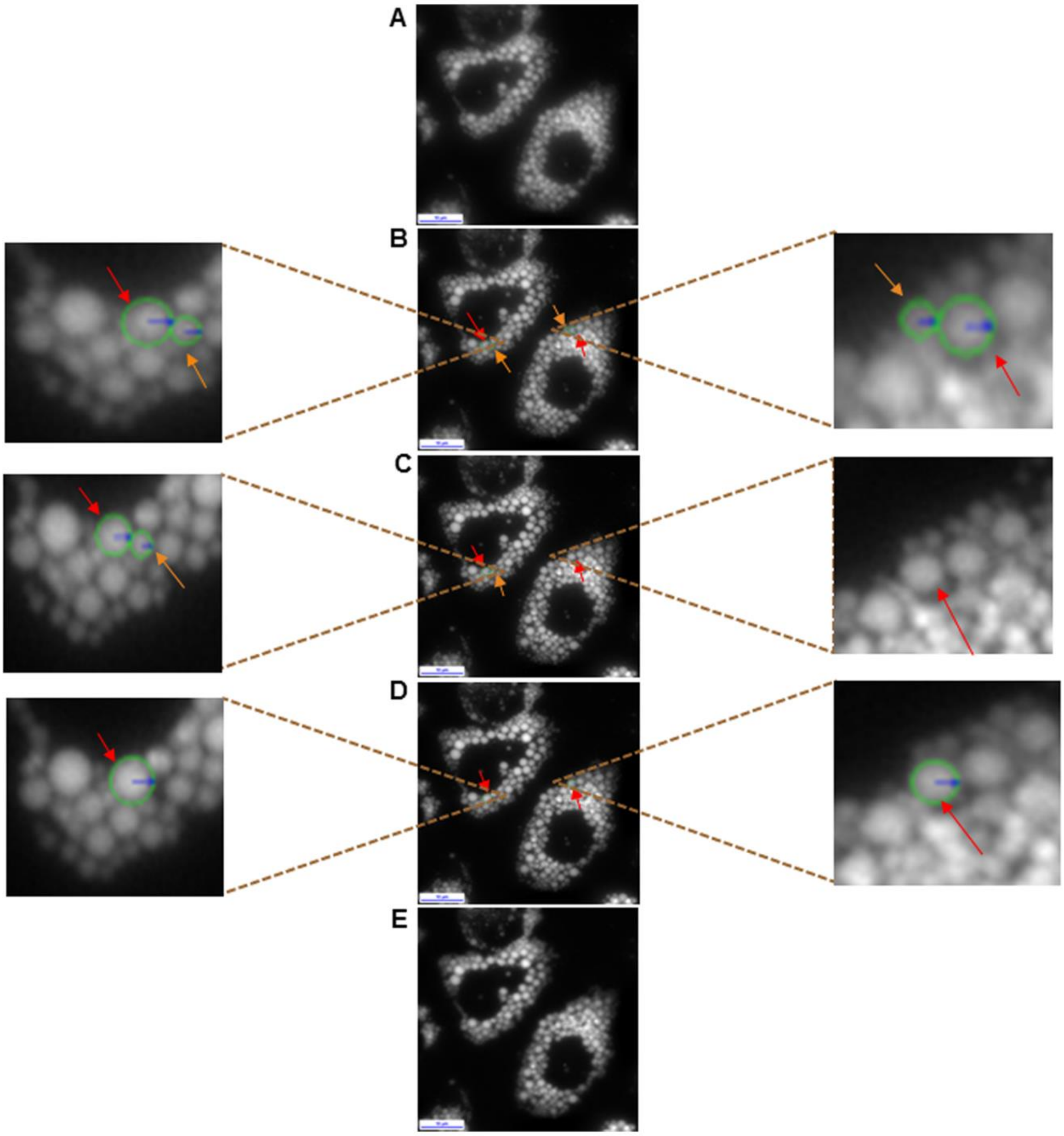

Live cell images of HuH7 cells treated with 0.5 mM of 1:1 mixture of oleic acid and palmitic acid for 24h. The smaller LDs (orange arrows) appear to fuse with larger LDs (red arrows). Panel B represents the initiation of fusion events where two LDs (v=0.86µm^3; v=2.9 µm^3) and LDs (v=1.49µm^3; v=6.88 µm^3) fuse to form a larger LD (v=3.2 µm^3) and (v=8.78µm^3), respectively (panel D). The figures were captured at 40x using time-lapse z-stack by phase contrast microscopy (Nikon TiE) and processed using the NIS elements software version 4.13. The lipid droplets were stained with the blue fluorescent MDP dye (images are not shown). The size bar refers to 10 µm.
