# Supplementary material for: Genomics of lipid-laden human hepatocyte cultures enables drug target screening for the treatment of non-alcoholic fatty liver disease
Source: BMC Med Genomics. 2018 Dec 14;11:111. doi: 10.1186/s12920-018-0438-7 (PMC6295111; doi:10.1186/s12920-018-0438-7)
Supplement: Supplementary file 4 — Figure S3. Live cell imaging of LD growth induced by fusogen. (A) Depicted are live cell images of HepG2 cells either (A1) without any treatment (K-) or (A2) treatment with the DMSO vehicle control. (A3) treatment with a 0.5 mM mixture of oleic acid and palmitic acid for 24 h; (A4) FA and 200 μM propranolol treatment for 3 h. The live cell images were captured at 40x using time-lapse z-stack by phase contrast fluorescence microscopy (Nikon TiE) and further analyzed using the NIS elements software version 4.13. (B) Depicted are live cell images of LD in HepG2 cells. Upon propranolol treatment the smaller LDs (orange arrows) appear to fuse with larger LDs (red arrows). The insets represent actively grown LDs. (B1) Clockwise: r = 9.14 μm, 12.79, 11.34, 7.9, 8.94, 9.04, 10.26, 11.38, 11.09, 10.28, 8.93. (B2) r = 12.41, 11.36, 12.46, 10.88; (B3) r = 16.33, 14.21, 11.11, 17.36; (B4) r = 19.25, 15.88, 19.54). (B5) The fusion events did not result in spherical lipid droplets; hence the volume could not be determined. The figures were captured at 40x using time-lapse phase contrast microscopy (Nikon TiE) and further processed using the NIS elements software version 4.13. The lipid droplets were stained with the blue fluorescent MDP dye (images are not shown). The size bar refers to 10 μm. (PDF 417 kb) [file 12920_2018_438_MOESM4_ESM.pdf]

Supplementary Figure S3: Live cell imaging of LD growth induced by fusogen.

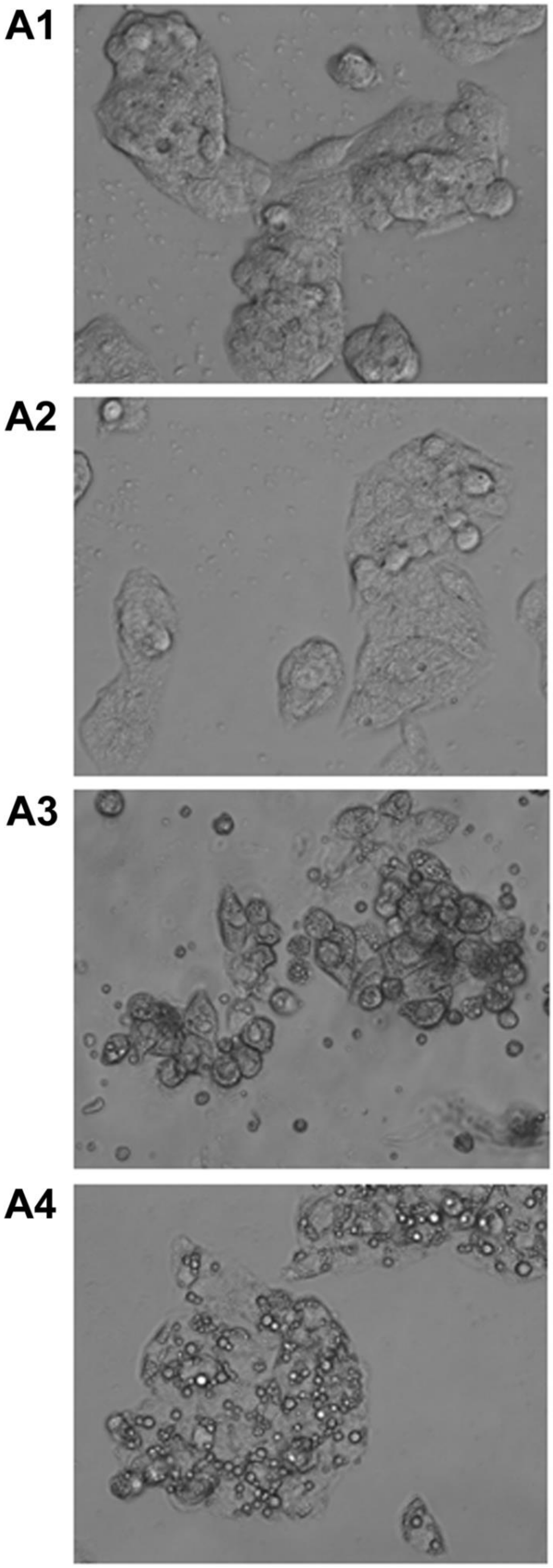

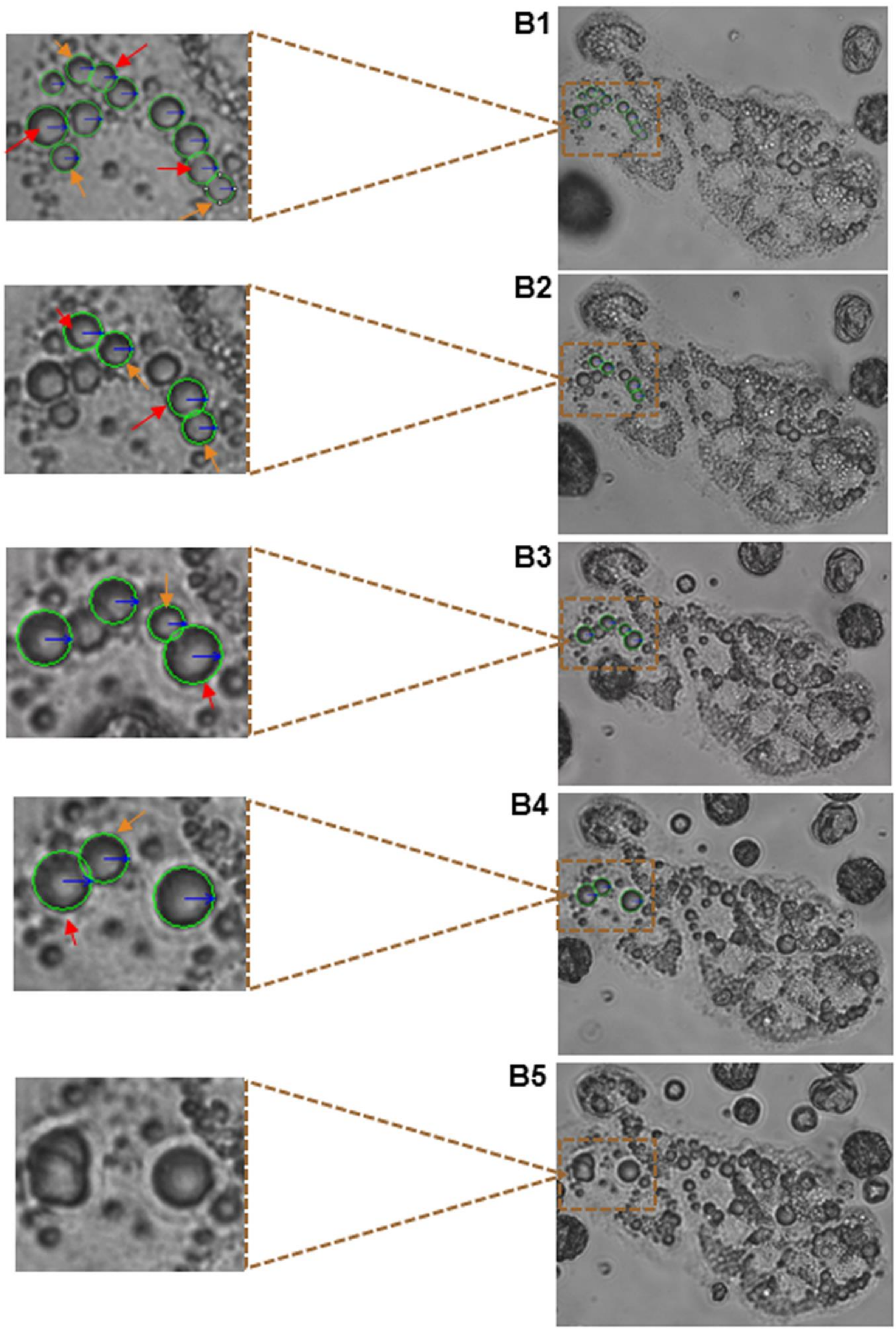
B1
B2
B3
B4
B5

(A) Depicted are live cell images of HepG2 cells either **(A1)** without any treatment (K-) or **(A2)** treatment with the DMSO vehicle control. **(A3)** treatment with a 0.5 mM mixture of oleic acid and palmitic acid for 24h; **(A4)** FA and 200 µM propranolol treatment for 3 hrs. The live cell images were captured at 40x using time-lapse z-stack by phase contrast fluorescence microscopy (Nikon TiE) and further analyzed using the NIS elements software version 4.13.

(B) Depicted are live cell images of LD in HepG2 cells. Upon propranolol treatment the smaller LDs (orange arrows) appear to fuse with larger LDs (red arrows). The insets represent actively grown LDs. **(B1)** Clockwise: r=9.14µm, 12.79, 11.34, 7.9, 8.94, 9.04, 10.26, 11.38, 11.09, 10.28, 8.93. **(B2)** r=12.41, 11.36, 12.46, 10.88; **(B3)** r=16.33, 14.21, 11.11, 17.36; **(B4)** r=19.25, 15.88, 19.54). **(B5)** The fusion events did not result in spherical lipid droplets; hence the volume could not be determined.

The figures were captured at 40x using time-lapse phase contrast microscopy (Nikon TiE) and further processed using the NIS elements software version 4.13. The lipid droplets were stained with the blue fluorescent MDP dye (images are not shown). The size bar refers to 10 µm.
