# Supplementary material for: Genomics of lipid-laden human hepatocyte cultures enables drug target screening for the treatment of non-alcoholic fatty liver disease
Source: BMC Med Genomics. 2018 Dec 14;11:111. doi: 10.1186/s12920-018-0438-7 (PMC6295111; doi:10.1186/s12920-018-0438-7)
Supplement: Supplementary file 5 — Table S2. List of mechanistically linked steatotic genes. (PDF 185 kb) [file 12920_2018_438_MOESM5_ESM.pdf]

Supplementary Table S2: List of mechanistically linked steatotic genes.

Lipogenesis	
ACACA	acetyl-CoA carboxylase alpha
ACACB	acetyl-CoA carboxylase beta
ACAT1	acetyl-CoA acetyltransferase 1
ACAT2	acetyl-CoA acetyltransferase 2
ACLY	ATP citrate lyase
AGPAT1	1-acylglycerol-3-phosphate O-acyltransferase 1
AGPAT2	1-acylglycerol-3-phosphate O-acyltransferase 2
AGPAT3	1-acylglycerol-3-phosphate O-acyltransferase 3
AGPAT5	1-acylglycerol-3-phosphate O-acyltransferase 5
AGPAT9	1-acylglycerol-3-phosphate O-acyltransferase 9
AGPS	alkylglycerone phosphate synthase
ANXA2	annexin A2
ANXA5	annexin A5
ANXA6	annexin A6
B4GALT5	UDP-Gal:betaGlcNAc beta 1,4- galactosyltransferase, 5
CREB1	cAMP responsive element binding protein 1
CREB3L3	cAMP responsive element binding protein 3-like 3
CREBL2	cAMP responsive element binding protein-like 2
DGAT1	diacylglycerol O-acyltransferase 1
DGAT2	diacylglycerol O-acyltransferase 2
ELOVL2	ELOVL fatty acid elongase 2
ELOVL3	ELOVL fatty acid elongase 3
ELOVL5	ELOVL fatty acid elongase 5
ELOVL6	ELOVL fatty acid elongase 6
FADS1	fatty acid desaturase 1
FADS2	fatty acid desaturase 2
FASN	fatty acid synthase
GALNT7	polypeptide N-acetylgalactosaminyltransferase 7
GNPAT	glyceronephosphate O-acyltransferase
GPAM	glycerol-3-phosphate acyltransferase,
GPAT2	glycerol-3-phosphate acyltransferase 2,
HMGB1	high mobility group box 1
INSIG1	insulin induced gene 1
INSIG2	insulin induced gene 2
LCAT	lecithin-cholesterol acyltransferase
LPIN2	lipin 2
MLXIPL	MLX interacting protein-like
MTOR	mechanistic target of rapamycin
NCEH1	neutral cholesterol ester hydrolase 1
PPAP2A	phosphatidic acid phosphatase type 2A
PPAP2B	phosphatidic acid phosphatase type 2AB
PPARG	peroxisome proliferator-activated receptor gamma
RHEB	Ras homolog enriched in brain
RPS6KA1	ribosomal protein S6 kinase, 90kDa, polypeptide 1

RPS6KA2	ribosomal protein S6 kinase, 90kDa, polypeptide 2
SCAP	SREBF chaperone
SCD	stearoyl-CoA desaturase (delta-9-desaturase)
SCD5	stearoyl-CoA desaturase 5
SERBP1	SERPINE1 mRNA binding protein 1
SPTLC1	serine palmitoyltransferase, long chain base subunit 1
SREBF1	sterol regulatory element binding transcription factor 1
TSC1	tuberous sclerosis 1
TSC2	tuberous sclerosis 2
UGCG	UDP-glucose ceramide glucosyltransferase
**Fatty acid oxidation/ mitochondrial stress**	
ACSL1	acyl-CoA synthetase long-chain family member 1
ACSL3	acyl-CoA synthetase long-chain family member 3
ACSL4	acyl-CoA synthetase long-chain family member 4
ACSL6	acyl-CoA synthetase long-chain family member 6
ASCL1	achaete-scute family bHLH transcription factor 1
CAT	catalse
CLIC1	chloride intracellular channel 1
CLIC4	chloride intracellular channel 4
CLIC6	chloride intracellular channel 6
CPT1A	carnitine palmitoyl transferase I
CPT2	carnitine palmitoyl transferase II
CYP24A1	cytochrome P450, family 24, subfamily A, polypeptide 1
CYP26A1	cytochrome P450, family 26, subfamily A, polypeptide 1
CYP26B1	cytochrome P450, family 26, subfamily B, polypeptide 1
DERL1	derlin 1
FAF2	Fas associated factor family member 2
FOXA1	forkhead box A1
FOXA2	forkhead box A2
HSDL2	Hydroxysteroid Dehydrogenase Like 2
MTTP	microsomal triglyceride transfer protein
PPARA	peroxisome proliferator-activated receptor alpha
PPARD	peroxisome proliferator-activated receptor beta or delta
SOD2	superoxide dismutase 2, mitochondrial
TUFM	Tu translation elongation factor, mitochondrial
**Lipid transport**	
**ABCA1**	ATP-binding cassette, sub-family A
AHR	aryl hydrocarbon receptor
AKT1	v-akt murine thymoma viral oncogene homolog 1
AKT2	v-akt murine thymoma viral oncogene homolog 2
AQP4	aquaporin 4
AQP7	aquaporin 7
CD36	CD36 molecule
CNR1	cannabinoid receptor 1
EGFR	epidermal growth factor receptor
FABP1	fatty acid binding protein 1
FABP6	fatty acid binding protein 6

FABP7	fatty acid binding protein 7
FAT1	FAT atypical cadherin 1
FATP2	Fatty acid transport protein 2
FATP5	Fatty acid transport protein 5
FXR1	farnesoid X receptor 1
FXR2	farnesoid X receptor 2
LDLR	low density lipoprotein receptor
NR1H3	nuclear receptor subfamily 1, group H, member 3
NR1H4	nuclear receptor subfamily 1, group H, member 4
NR1I2	nuclear receptor subfamily 1, group I, member 2
NR5A2	nuclear receptor subfamily 5, group A, member 2
RXRA	retinoid X receptor, alpha
RXRB	retinoid X receptor, beta
RXRG	retinoid X receptor, gamma
SLC27A2	solute carrier family 27 (fatty acid transporter), member 2
SLC27A5	solute carrier family 27 (fatty acid transporter), member 5
THR	thyrotropin-releasing hormone
**Biomarkers**	
COL4A4	collagen, type IV, alpha 4
COL4A6	collagen, type IV, alpha 6
FGF21	fibroblast growth factor 21
GGT1	gamma-glutamyltransferase
GLUD1	glutamate dehydrogenase 1
GLUD2	glutamate dehydrogenase 2
GOT1	glutamic-oxaloacetic transaminase 1
GPT	glutamic-pyruvate transaminase
GSTA1	glutathione S-transferase 1
KRT18	keratin 18
KRT8	keratin 8
LAMA1	laminin, alpha
LAMB1	laminin, beta
LAMC1	laminin, gamma
LDHA	lactate dehydrogenase A
MIR122	microRNA 122
TIMP1	TIMP metallopeptidase inhibitor 1
TXN1	thioredoxin 1
TXN2	thioredoxin 2
**LD growth/ER stress**	
ABHD5	abhydrolase domain containing 5
ABHD6	abhydrolase domain containing 6
ABHD12	abhydrolase domain containing 12
ACTB	actin, beta
APOB	apolipoprotein B
APOE	apolipoprotein E
ARFGAP1	ADP-ribosylation factor GTPase activating protein 1
ATG5	autophagy related 5
ATG7	autophagy related 7

CAV1	Caveolin1
CAV2	Caveolin2
CIDEB	cell death-inducing DFFA-like effector b
CIDEC	cell death-inducing DFFA-like effector c
DNAAF1	dynein, axonemal, assembly factor 1
DNAH3	Dyenin
DST	dystonin
DYNC1H1	Dyenin
DYNC1I1	Dyenin
DYNC2H1	Dyenin
DYNLL1	dynein, light chain, LC8-type 1
DYNLL2	dynein, light chain, LC8-type 2
ERLIN1	ER lipid raft associated 1
ERLIN2	ER lipid raft associated 2
ERP29	endoplasmic reticulum protein 29
FITM1	fat storage-inducing transmembrane protein 1
FITM2	fat storage-inducing transmembrane protein 2
FLOT1	flotillin 1
HILPDA	hypoxia inducible lipid droplet-associated
KIF1A	Kinesin
LIPE	lipase, hormone-sensitive
LPL	lipoprotein lipase
MAP1LC3B	microtubule-associated protein 1 light chain 3 beta
MGLL	monoglyceride lipase
NAPA	N-ethylmaleimide-sensitive factor attachment protein, alpha
NSF	N-ethylmaleimide-sensitive factor
PDIA3	protein disulfide isomerase family A, member 3
PKA	protein kinase, cAMP-dependent, catalytic, alpha
PKD	Protein kinase D
PLD1	Phospholipase D1
PLIN1	Perilipin 1
PLIN2	Perilipin 2
PLIN3	Perilipin 3
PLIN4	Perilipin 4
PLIN5	Perilipin 5
PNPLA2	patatin-like phospholipase domain containing 2
PNPLA3	patatin-like phospholipase domain containing 3
RAB11A	Rab family protein 11A
RAB18	Rab family protein 18
RAB32	Rab family protein 32
RAB35	Rab family protein 35
RAB5A	Rab family protein 5A
RAB7A	Rab family protein 7A
SEC22A	SEC22 vesicle trafficking protein homolog A
SEC22B	SEC22 vesicle trafficking protein homolog B
SEC22C	SEC22 vesicle trafficking protein homolog C
SNAP23	synaptosomal-associated protein, 23kDa

SNAP25	synaptosomal-associated protein, 25kDa
SNCA	synuclein, alpha
SNCG	synuclein, gamma
STX5	syntaxin 5
TUBB	tubulin, beta class I
TUBB2A	tubulin, beta 2A class IIa
USP20	ubiquitin specific peptidase 20
USP33	ubiquitin specific peptidase 33
VAMP2	vesicle-associated membrane protein 2
VAMP3	vesicle-associated membrane protein 3
VAMP4	vesicle-associated membrane protein 4
VIM	vimentin
VTI1B	vesicle transport through interaction with t-SNAREs 1B
**Inflammation and signalling events**	
ARG1	arginase 1
ARG2	arginase 2
CCL2	chemokine (C-C motif) ligand 2
CCR2	chemokine (C-C motif) receptor 2
CEBPA	CCAAT/enhancer binding protein, alpha
CRP	C-reactive protein
CTSD	cathepsin D
CTSS	cathepsin S
CXCL1	chemokine (C-X-C motif) ligand 1
CXCL2	chemokine (C-X-C motif) ligand 2
CXCL3	chemokine (C-X-C motif) ligand 3
CXCL6	chemokine (C-X-C motif) ligand 6
CXCL9	chemokine (C-X-C motif) ligand 9
CXCL10	chemokine (C-X-C motif) ligand 10
CXCL13	chemokine (C-X-C motif) ligand 13
CXCL14	chemokine (C-X-C motif) ligand 14
EIF2AK2	eukaryotic translation Initiation Factor 2alpha
EIF2AK3	eukaryotic translation Initiation Factor alpha, kinase3
EIF2B1	eukaryotic translation initiation factor 2B, 1 alpha
EIF2S1	eukaryotic translation initiation factor 2, 1 alpha
EIF2S3	eukaryotic translation initiation factor 2, 3 gamma
HIF1A	hypoxia inducible factor 1, alpha subunit
IL1A	interleukin 1A
IL6	interleukin 6
IL8	interleukin 8
IL17A	interleukin 17A
IL18	interleukin 18
IL10	interleukin 10
IL12A	interleukin 12A
IL32	interleukin 32
JAK1	Janus kinase 1
JAK2	Janus kinase 2

JAK3	Janus kinase 3
MAPK1	mitogen-activated protein kinase 1
MAP2K2	mitogen-activated protein kinase kinase 2
MAPK8	mitogen-activated protein kinase 8
MAPK9	mitogen-activated protein kinase 9
MAPK10	mitogen-activated protein kinase 10
NFKB1	nuclear factor of kappa light polypeptide gene enhancer in Bcells1
NOS2	nitric oxide synthase 2, inducible
PEBP1	phosphatidylethanolamine binding protein 1
PI3KC2A	phosphatidylinositol-4-phosphate 3-kinase, type 2 alpha
PRDX6	peroxiredoxin 6
PRKAA1	protein kinase, AMP-activated, alpha 1 catalytic subunit
PRKAA2	protein kinase, AMP-activated, alpha 2 catalytic subunit
PRKAB1	protein kinase, AMP-activated, beta 1 non-catalytic subunit
PRKAB2	protein kinase, AMP-activated, beta 2 non-catalytic subunit
PRKACA	protein kinase, cAMP-dependent, catalytic, alpha
PRKAG1	protein kinase, AMP-activated, gamma 1 non-catalytic subunit
PRKAG2	protein kinase, AMP-activated, gamma 2 non-catalytic subunit
PRKAG3	protein kinase, AMP-activated, gamma 3 non-catalytic subunit
PRKD1	protein kinase D1
PTEN	phosphatase and tensin homolog
PTGER2	prostaglandin E receptor 2
RHOA	ras homolog family member A
RHOB	ras homolog family member B
ROCK1	Rho-associated, coiled-coil containing protein kinase 1
ROCK2	Rho-associated, coiled-coil containing protein kinase 2
SERPINE1	serpin peptidase inhibitor, clade E member 1
SERPINE2	serpin peptidase inhibitor, clade E member 2
SOCS1	suppressor of cytokine signaling 1
SOCS2	suppressor of cytokine signaling 2
SOCS3	suppressor of cytokine signaling 3
STAT1	signal transducer and activator of transcription 1
STAT3	signal transducer and activator of transcription 3
STAT4	signal transducer and activator of transcription 4
STAT5A	signal transducer and activator of transcription 5A
STAT5B	signal transducer and activator of transcription 5B
STIP1	stress-induced phosphoprotein 1
TGFB1	transforming growth factor, beta 1
TGFB2	transforming growth factor, beta 2
TGFBR2	transforming growth factor, beta receptor II
TLR4	toll-like receptor 4
TNFA	tumor necrosis factor a
TNFAIP2	tumor necrosis factor, alpha-induced protein 2
TNFAIP8	tumor necrosis factor, alpha-induced protein 8
UBXN4	UBX domain protein 4
UCP2	uncoupling protein 2
VCAN	versican

Glucose metabolism	
ANGPTL4	angiopoietin-like 4
CES1	carboxylesterase 1
ENO1	Enolase1
ENO3	Enolase3
FOXO1	forkhead box O1
GK	glycerol kinase
HNF4A	hepatocyte nuclear factor 4, alpha
IRS1	Insulin receptor substrate1
IRS2	Insulin receptor substrate2
PDK4	Pyruvate dehydrogenase kinase, isozyme 4
PGAM1	phosphoglycerate mutase 1
PKLR	pyruvate kinase
SLC2A1	solute carrier family 2, member 1
SLC2A10	solute carrier family 2, member 10
SLC2A5	solute carrier family 2, member 5
TXNIP	thioredoxin interacting protein
