# Supplementary material for: Genomics of lipid-laden human hepatocyte cultures enables drug target screening for the treatment of non-alcoholic fatty liver disease
Source: BMC Med Genomics. 2018 Dec 14;11:111. doi: 10.1186/s12920-018-0438-7 (PMC6295111; doi:10.1186/s12920-018-0438-7)
Supplement: Supplementary file 6 — Table S3. Compilation of NAFLD responsive genes in in-vitro/in-vivo comparisons. (PDF 96 kb) [file 12920_2018_438_MOESM6_ESM.pdf]

Supplementary Table S3: Compilation of NAFLD responsive genes in *in vitro* / *in vivo* comparisons.

Condition	***Gene***
HepG2_72h, HepG2_7D, Huh7_72h, NAFLD_Patients, PHH_72h	PDK4
HepG2_72h, Huh7_72h, NAFLD_Patients, PHH_72h	FOXO3, DAB2
HepG2_72h, HepG2_7D, NAFLD_Patients, PHH_72h	AASS, KLB
HepG2_7D, Huh7_72h NAFLD_Patients, PHH_72h	BRWD1, CPT1A, CYP26A1, PLIN2
HepG2_72h, HepG2_7D, Huh7_72h, NAFLD_Patients	C1S, FUS, ITGA1, MAP3K8, HNRNPD, TNFSF10
HepG2_72h, HepG2_7D, Huh7_72h, PHH_72h	HSDL2, KLF11
HepG2_72h, NAFLD_Patients, PHH_72h	PRPF4B, THSD4, AKAP12, CYP26B1, SMARCA2
Huh7_72h, NAFLD_Patients, PHH_72h	COL7A1, PLAGL1, ASNS, CIRBP, TSR1, DLAT, GAS5, GDF15, BEX2, TNRC6A, PTPRF, TFPI, HIST1H2BD, TMEM107, RSL1D1, COL27A1, MPZL2, NUPL1, DNAJC3, TNFAIP8L1, ILF3, TXLNA, SEPP1, AP3D1, ABLIM3
HepG2_7D, NAFLD_Patients, PHH_72h	N4BP2L1, EPB41L5
HepG2_72h, Huh7_72h, NAFLD_Patients	N4BP2L2, CPEB3, MAD2L1, BRD4, FH, PFKFB2, API5, NAB1, C2orf15, LRG1, ST6GAL1, SOX4, FAM102B, STOX2, SSR3, TMPO, NAA50, OXR1, LRPPRC, TFF2, IFRD1, GFM2, ID4, CYP3A7, LARP1B, SPARC, BIRC3, TMCO3, MSH6, KIAA1704, EIF3M, HINT3, PICALM, NAV2, NR2F2, NUCKS1, SLC2A14, PGF, LIPG, SAA1, RAB18, NXT2, CEP70, RFC3, FAM208B, ZNF124, GFM1, ZBTB8A, RNF207, SNAP23, SLC1A4, RPAIN, RECK, STK4, VAMP3, PRLR, AKAP13, RAB27A, ETNK1, PXK

HepG2_72h, HepG2_7D, NAFLD_Patients	HSPD1, SKAP2, SULF2, NPNT, ANXA2, ZDHHC2, HMGB2, SLC38A1, SLC38A4, GABBR1, IL13RA1, ENPP2, LRRC8E, EXT1, MPP5, LOXL4, AGXT, DLK1, RGMB, TGM2, SEC16B, ERAP1, EPB41L1, PHF17, PPARA, SRPK2, KLHDC10, CEP152
HepG2_7D, Huh7_72h, NAFLD_Patients	ACSM3, SHROOM1, HS2ST1, CEP120, AKIRIN1, LDLR, DNAJB4, NT5E, LGALS2, ZBTB21, SYNE1, MCM4, GALT, AJUBA, RPL31, MRPL4, MBNL2, ABCC3, SCD, LYN, DUSP6, MND1, KCTD6, FMO5, RIT1, AGMAT, CXADR, CDC6, ITPRIPL2, FAM126B, AFG3L1P, RHOU, CPSF6, LUC7L3, RAPGEF2, STXBP1, RUFY3, DNAJB1, ELF3, TUFT1, DENR, FOXA1, DDIT3, RHOB, SPIRE1, FGFR1OP2, MGAT4A, PDS5A, SCAF11, SLC38A7
HepG2_72h, Huh7_72h, PHH_72h	TRMT1
HepG2_72h, HepG2_7D, PHH_72h	HIST1H4A, ZNF507
HepG2_7D, Huh7_72h, PHH_72h	ACADVL, MBNL3, FAXDC2, ALMS1, TAOK1, CYP24A1
HepG2_72h, HepG2_7D, Huh7_72h	PHF20, NFE2L3, CPEB4, SERPINA3, FANCI, TMEM198, TMEM140, MTFR2, BCCIP

The genes given in Table S2 refer to the Venn diagram of Figure 5C.
